# Information Metamaterial Systems

**DOI:** 10.1016/j.isci.2020.101403

**Published:** 2020-07-23

**Authors:** Tie Jun Cui, Lianlin Li, Shuo Liu, Qian Ma, Lei Zhang, Xiang Wan, Wei Xiang Jiang, Qiang Cheng

**Affiliations:** 1 State Key Laboratory of Millimeter Waves, Southeast University, Nanjing 210096, China; 2 State Key Laboratory of Advanced Optical Communication Systems and Networks, Department of Electronics, Peking University, Beijing 100871, China

**Keywords:** Electromagnetic Waves, Information Systems, Metamaterials

## Abstract

Metamaterials have great capabilities and flexibilities in controlling electromagnetic (EM) waves because their subwavelength meta-atoms can be designed and tailored in desired ways. However, once the structure-only metamaterials (i.e., passive metamaterials) are fabricated, their functions will be fixed. To control the EM waves dynamically, active devices are integrated into the meta-atoms, yielding active metamaterials. Traditionally, the active metamaterials include tunable metamaterials and reconfigurable metamaterials, which have either small-range tunability or a few numbers of reconfigurability. Recently, a special kind of active metamaterials, digital coding and programmable metamaterials, have been presented, which can realize a large number of distinct functionalities and switch them in real time with the aid of field programmable gate array (FPGA). More importantly, the digital coding representations of metamaterials make it possible to bridge the digital world and physical world using the metamaterial platform and make the metamaterials process digital information directly, resulting in information metamaterials. In this review article, we firstly introduce the evolution of metamaterials and then present the concepts and basic principles of digital coding metamaterials and information metamaterials. With more details, we discuss a series of information metamaterial systems, including the programmable metamaterial systems, software metamaterial systems, intelligent metamaterial systems, and space-time-coding metamaterial systems. Finally, we introduce the current progress and predict the future trends of information metamaterials.

## Graphical Abstract

**Figure:**
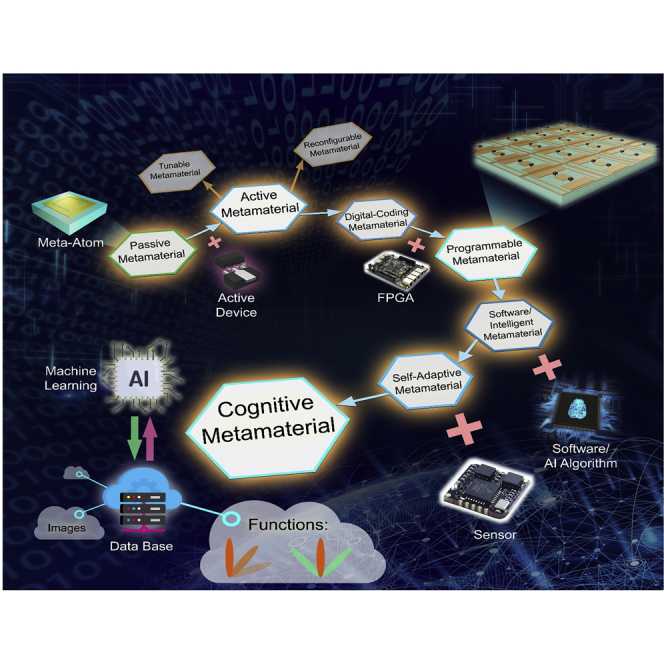

## Introduction

### Evolution of Metamaterials

**Figure 1:**
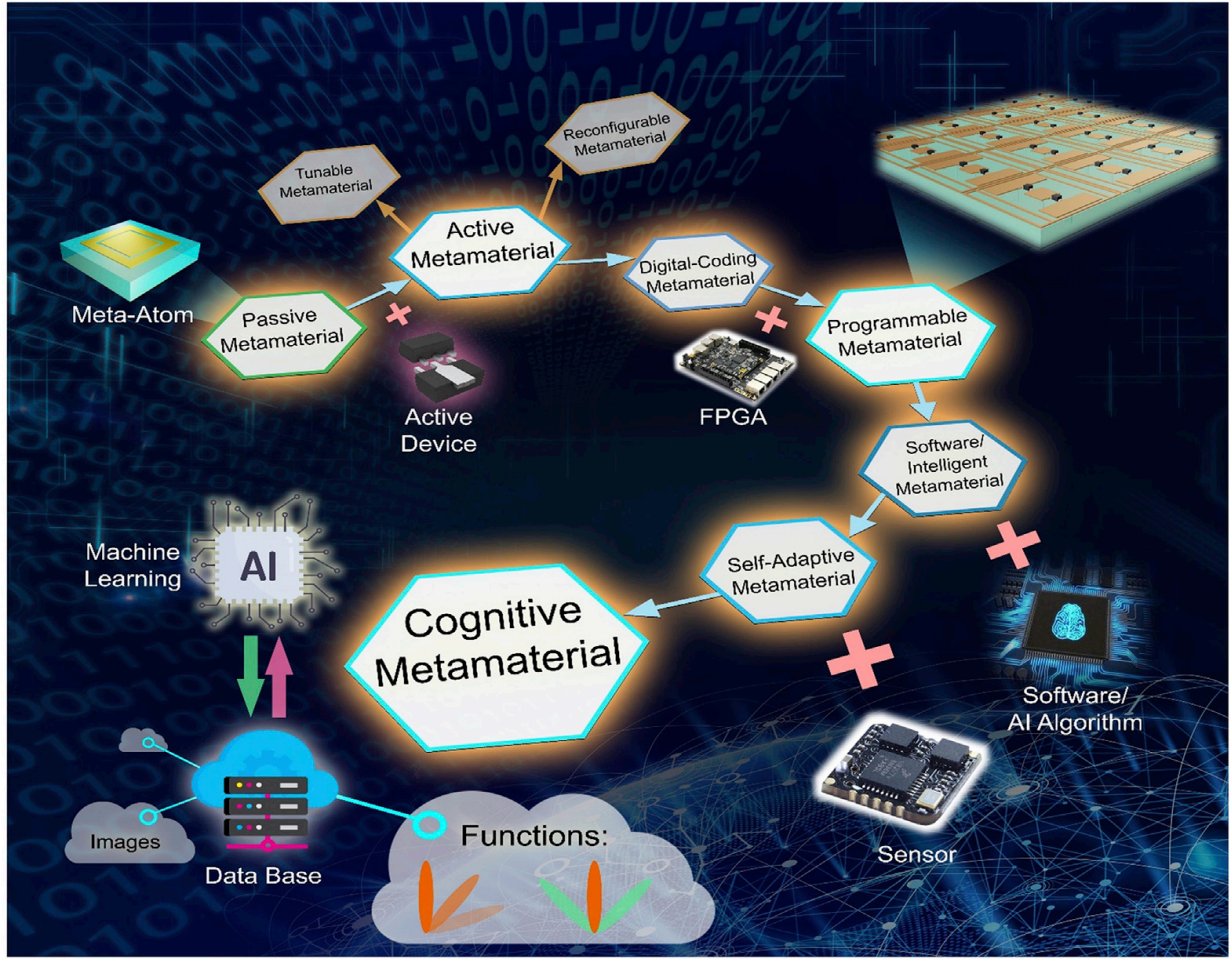
Evolution of Metamaterials By integrating various electronic devices and machine learning algorithms, the metamaterial functions have been greatly enriched by developing the digital coding metamaterial, programmable metamaterial, software/intelligent metamaterial, self-adaptive metamaterial, and cognitive metamaterial.

Since Sir John Pendry proposed to realize negative permittivity using periodic structure of thin wires in 1996 (Pendry et al., 1996), modern metamaterials (including metasurfaces, the same in the remaining contents) have received great progress in the past 20 years and are still in the frontiers of physics, chemistry, material, and information societies. Many new findings, devices, and even systems have been presented in this area. From the perspective of achievable functions, the development of metamaterial is classified into four stages. The first stage is for passive metamaterials, which are composed of specially designed artificial structures in periodic or nonperiodic arrays of subwavelength unit cells (or called as meta-atoms, see Figure 1) to reach homogeneous or inhomogeneous effective medium parameters that do not exist in nature or are difficult to achieve in practice. The passive metamaterials have been well developed in both microwave and optical frequency bands, showing the powerful capability to control electromagnetic (EM) waves in desired ways and reaching a series of fantastic physical phenomena and useful devices (Smith et al., 2000, 2005; Pendry, 2000; Shelby et al., 2001; Valentine et al., 2008; Fang et al., 2005; Pendry et al., 2006; Schurig et al., 2006; Li and Pendry, 2008; Liu et al., 2009; Ma and Cui, 2010; Ergin et al., 2010; Lai et al., 2009; Jiang et al., 2011; Cheng et al., 2009; Ma & Cui, 2010, 2010; Chen et al., 2011; Wan et al., 2014; Kuester et al., 2003; Falcone et al., 2004; Holloway et al., 2005, 2012; Yu et al., 2011; Ni et al., 2012, 2013; Yu and Capasso, 2014; Aieta et al., 2012; Huang et al., 2012; Mehmood et al., 2016; Yin et al., 2013; Luo et al., 2015; Grady Heyes et al., 2013).

The earlier study of metamaterials has focused on homogeneous situations with extreme effective medium parameters (e.g. negative permittivity, negative permeability, and zero index of refraction) to explore unusual physical phenomena. The first well-known phenomenon is negative refraction, which was created by a so-called left-handed material with negative permittivity and negative permeability simultaneously (Smith et al., 2000; Pendry, 2000; Shelby et al., 2001; Valentine et al., 2008). This special metamaterial can also bring in the exciting perfect lens and super lenses (Pendry, 2000; Fang et al., 2005). However, the homogeneous metamaterials have limited capabilities in controlling the EM waves. To change the situation, transformation optics theory was established by Sir John Pendry in 2006 (Pendry et al., 2006), from which the EM waves can be manipulated in arbitrarily desired ways, resulting in many exciting phenomena, such as invisibility cloaking (Schurig et al., 2006; Li and Pendry, 2008; Liu et al., 2009; Ma and Cui, 2010; Ergin et al., 2010) and optical illusions (Lai et al., 2009; Jiang et al., 2011). Usually, the transformation optics results in anisotropic and inhomogeneous metamaterials with extreme medium parameters, which are hard to realize in practice. By using certain approximations, some transformation-optics devices (e.g. invisibility cloaks, EM black holes, and microwave illusions) have been demonstrated experimentally (Schurig et al., 2006; Liu et al., 2009; Ma and Cui, 2010; Ergin et al., 2010; Jiang et al., 2011). However, such kinds of metamaterial devices cannot be used in engineering application owing to narrow bands, high losses, or large volumes. Later, another kind of metamaterial—gradient-index material—has been proposed for realizing Luneburg lens, flattened Luneburg lens, and slab lenses (Smith et al., 2005; Cheng et al., 2009; Ma and Cui, 2010; Chen et al., 2011; Wan et al., 2014), which have good performance of wide-frequency band and small loss and have found applications as microwave antennas.

In many situations, the application scope of the three-dimensional (3D) metamaterials is limited due to their large volumes. Then two-dimensional (2D) versions of metamaterials, the metasurfaces, have attracted great attention owing to their planar geometrical structures and easy fabrications (Kuester et al., 2003; Falcone et al., 2004; Holloway et al., 2005, 2012; Yu et al., 2011; Ni et al., 2012; Yu and Capasso, 2014; Aieta et al., 2012; Huang et al., 2012; Mehmood et al., 2016; Yin et al., 2013; Luo et al., 2015; Grady Heyes et al., 2013). By designing gradient phase shifts periodically on a metasurface, one can achieve anomalous reflection and refraction when the EM wave is incident to the metasurface, which are governed by the generalized Snell's laws of reflection and refraction (Holloway et al., 2012). Since 2011, the gradient-phase metasurfaces have been intensively investigated and used in realizing optical vortex beams (Huang et al., 2012; Mehmood et al., 2016), photonic spin Hall effect (Yin et al., 2013; Luo et al., 2015), polarization controls (Ni et al., 2013), holograms (Chen et al., 2014; Zheng et al., 2015), metalenses (Khorasaninejad et al., 2016), and spatial wave to surface wave conversions (Sun et al., 2012). However, no matter it is a 3D version or 2D version, once a structure-only passive metamaterial is fabricated, its functionality will be fixed.

To realize dynamic manipulations of EM waves, the passive metamaterials have to integrate active devices to reach active metamaterials (see Figure 1), which is the second stage of the metamaterial development. In the active metamaterials, the unit cells consist of meta-atoms and active devices (e.g. PIN diodes, varactors, amplifiers, semiconductors, micro-fluids, and VO_2_) to change their EM responses under the external excitations (Chen et al., 2006, 2017; Cong et al., 2017; Liu et al., 2014, Liu et al., 2016a, Liu et al., 2016, 2016c, 2016d, 2016e; Yao et al., 2014; Lee et al., 2014; Miao et al., 2015; Gutruf et al., 2016; Zhao et al., 2018; Wang et al., 2015, 2019; Zhu et al., 2015; Oliveri et al., 2015; Pitchappa et al., 2016; Huang et al., 2017a; Huang et al., 2017b). Traditionally, the active metamaterials include tunable metamaterials and reconfigurable metamaterials (see Figure 1). As the first and simple active metamaterials, the tunable metamaterials usually indicate to realize some similar functions (such as shifting the resonance peaks and perfect absorptions) by tuning the active devices (Liu et al., 2014, Liu et al., 2016a, Liu et al., 2016, 2016c, 2016d, 2016e; Yao et al., 2014; Lee et al., 2014; Miao et al., 2015; Gutruf et al., 2016; Zhao et al., 2018; Wang et al., 2019). Although the reconfigurable metamaterials can exhibit significantly different functions (such as changing the polarization states and controlling the working bandwidth) by switching the active devices, the number of functions is very limited (Wang et al., 2015; Zhu et al., 2015; Oliveri et al., 2015; Pitchappa et al., 2016; Huang et al., 2017a; Huang et al., 2017b; Chen et al., 2017). Besides, how to tune and switch different states of the tunable metamaterials and reconfigurable metamaterials in ***real time*** is the other difficulty.

As the counterpart of analog circuits, the traditional passive, tunable, and reconfigurable metamaterials can be regarded as analog metamaterials. The digital coding representation of meta-atom makes it possible to realize digital metamaterials (Cui et al., 2014). In fact, the digital metamaterial is a branch of active metamaterials (see Figure 1), in which the controlling state of the active device is discretized to 2, 4, or 8 states to achieve the digital states of the meta-atom for 1-bit coding (two digital states 0 and 1 with 180° phase difference), 2-bit coding (four digital states 00, 01, 10, and 1 with 90° phase difference), and 3-bit coding (eight digital states 000, 001, 010, 011, 100, 101, 110 and 111 with 45° phase difference), respectively (Cui et al., 2014). It was shown that the EM waves are fully controlled by the spatial coding sequence on the metamaterial (Cui et al., 2014, 2016, 2017; Liu et al., 2016a, Liu et al., 2016, 2016c, 2016d, 2016e; Li et al., 2019a, 2019b; Shannon, 2001; Wu et al., 2018, Wu et al., 2019; Zhang et al., 2017). Hence one can design many sets of digital coding sequences on the metamaterial aperture (in fact, there are 2^*N*^ sets of coding sequences for a 1-bit digital coding metamaterial containing *N* meta-atoms), calculate their corresponding functions, and store them in a field programmable gate array (FPGA) (Cui et al., 2014). Integrating the digital coding metamaterial with FPGA will yield a programmable metamaterial (Cui et al., 2014), as illustrated in Figure 1. This is the third stage of metamaterial developments.

Making the metamaterial be field programmable is a big progress in the developments of metamaterials because of the following features:

• A single programmable metamaterial can accomplish many significantly distinct functions (e.g. single-beam radiation, different multi-beam radiations, beam scanning, wave diffusion, and vortex beam generation) (Cui et al., 2014, 2017; Liu et al., 2016a, Liu et al., 2016, 2016c, 2016d, 2016e; Li et al., 2019a, 2019b; Shannon, 2001);
• All these functions are switched **in real time** by changing the digital states and sending instructions by FPGA (Cui et al., 2014; Li et al., 2019a, 2019b);
• The digital coding metamaterial builds up a bridge between the physical world and the digital world (Wan et al., 2016, 2019; Shuang et al., 2020; Zhang et al., 2018a, 2018b), which helps establish new information systems, pushing the metamaterials to system-level applications (Zhang et al., 2020a, Zhang et al., 2020b, 2020c; Lipworth et al., 2013; Hunt et al., 2013; Li et al., 2016; Wang et al., 2016; Li et al., 2018; Li et al., 2017; Cui et al., 2019; Li et al., 2019a, 2019b; Li, 2019; Li et al., 2020; Zhao et al., 2019; Dai et al., 2018; Dai et al., 2019; Tang et al., 2019a, 2019b; Dai et al., 2020; Zhang et al., 2018a, 2018b; Zhang et al., 2019a, 2019b; Hadad et al., 2015; Shaltout et al., 2015; Hadad et al., 2016; Correas-Serrano et al., 2016).

For example, the reprogrammable holographic imaging system (Zhang et al., 2020a), single-sensor and single-frequency microwave imaging system (Zhang et al., 2020b), and new architecture wireless communication system (Lipworth et al., 2013) have been developed with the programmable metamaterials. More importantly, computer codes, software, and machine-learning algorithms are easy to be integrated with the programmable metamaterials in generating software metamaterials (Li, 2019; Li et al., 2020; Zhao et al., 2019; Dai et al., 2018, 2019; Zhang et al., 2020c) and intelligent metamaterials (Tang et al., 2019a; 2019b; Dai et al., 2020; Zhang et al., 2018a, 2018b; Zhang et al., 2019a, 2019b), as shown in Figure 1. Based on the intelligent metamaterial, a microwave camera was presented, which can accomplish the overall imaging of a whole scene (such as a home), focusing the EM beam to a target (such as human being), and identifying the actions (such as hand signs and breath) by using three deep-learning algorithms (Shaltout et al., 2015).

In this review article, we firstly make a brief introduction on the concepts and principles of the digital coding metamaterials, programmable metamaterials, and information metamaterials and then present the information metamaterial systems intensively, including the programmable metamaterial systems, software metamaterial systems, intelligent metamaterial systems, and space-time-coding digital metamaterial systems. Finally, we introduce the currently exciting progress on self-adaptively smart metamaterial (Hadad et al., 2016), which can be regarded as the fourth stage of metamaterial developments due to its automatic decision-making ability, and future directions of the information metamaterials.

## Concepts and Basic Principles of Information Metamaterials

### Digital Coding Metamaterials

**Figure 2:**
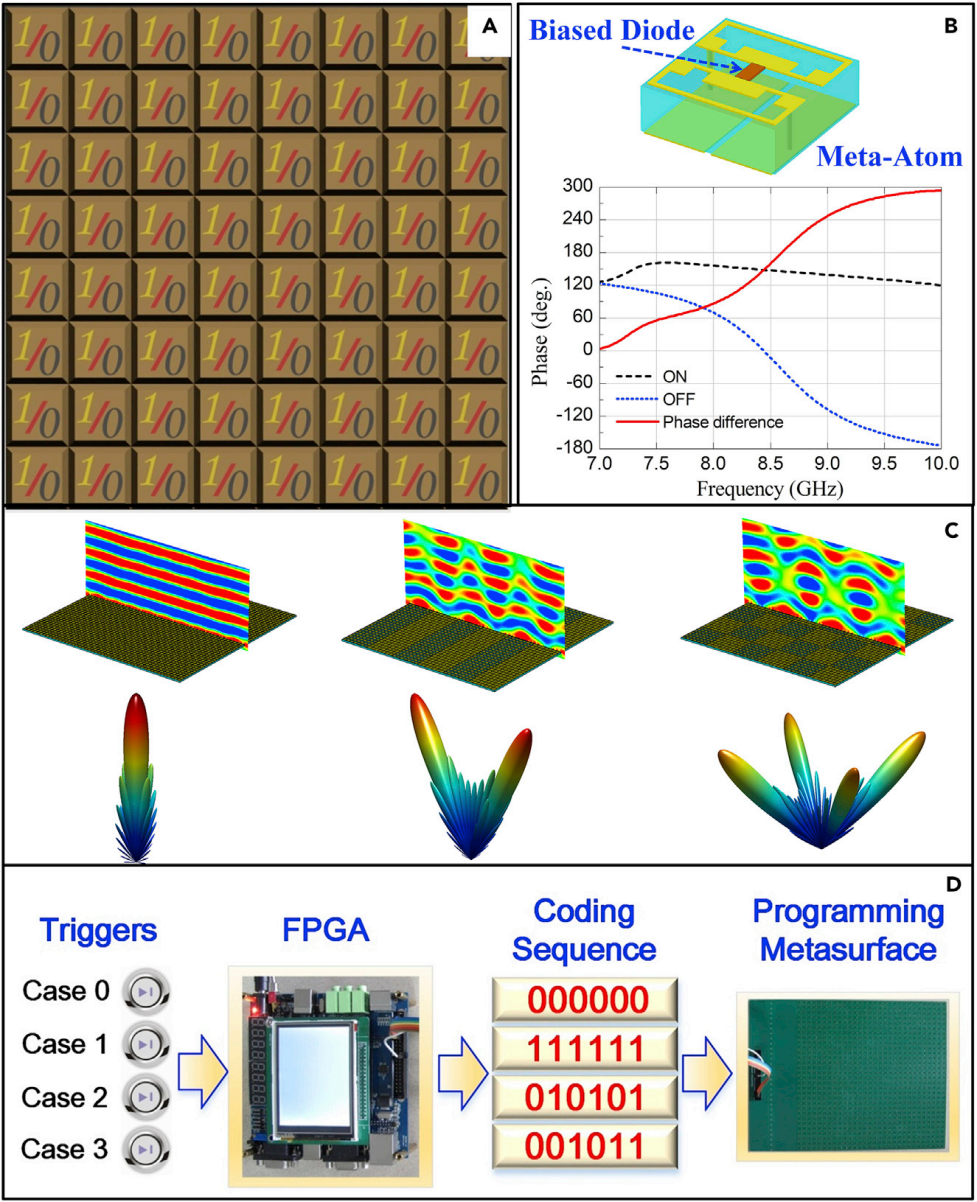
The Digital Coding Metamaterials and Their Constitutive Digital Meta-Atoms (A) The 1-bit digital coding metamaterial composed of two types of meta-atoms with “0” and “1” states. (B) Top: the structure of a 1-bit digital meta-atom, which behaves as the digital states “0” and “1” when the biased PIN diode is switched “OFF” and “ON,” respectively; bottom: the corresponding phase responses of the 1-bit digital meta-atom as the PIN diode is switched “OFF” and “ON” over a range of frequencies. (C) The theoretical and full-wave simulation results of the 1-bit digital coding metamaterials with different coding sequences under the normal incidence of EM waves. (D) The configuration and its working mechanism of the programmable metamaterial. Adapted from (Cui et al., 2014).

In 2014, Cui et al. proposed the concepts of digital coding metamaterials (Cui et al., 2014), which were designed to control the EM waves in a digitally discretized manner. As a branch of metamaterials, the digital coding metamaterials utilize different coding sequences to manipulate the reflected or transmitted wavefronts (Liu et al., 2016a, Liu et al., 2016, 2016c, 2016d, 2016e). The digital representation of the metamaterials has advantages of simplifying the design and optimization process, which helps revisit metamaterials from the perspective of information science and bridge the physical world and the digital world (Cui et al., 2017). For example, a 1-bit digital coding metamaterial is constructed by two distinct digital meta-atoms “0” and “1” with 180° phase difference, as illustrated in Figure 2A. By integrating active elements with the digital meta-atoms, their coding states can be switched in real time. The top of Figure 2B displays the structure of a 1-bit digital meta-atom (Cui et al., 2014), which behaves as digital states “0” and “1” when the biased PIN diode is switched “OFF” and “ON,” respectively. The bottom of Figure 2B demonstrates the corresponding reflection phases of the 1-bit digital meta-atom under the two states, from which we can see that the phase difference reaches 180° at the designed frequency of 8.6 GHz.

The concept of the digital coding metamaterial can be extended from 1-bit coding to multi-bit coding. For instance, the structure of a 2-bit digital meta-atom is integrated with three PIN diodes (Li et al., 2019a, 2019b) and has four different phase responses to define the 2-bit digital states “00”, “01”, “10,” and “11” with the phase difference of 90°. Similarly, higher-bit digital meta-atoms can be defined and realized. In contrast to the traditional metamaterials that are controlled by the effective medium parameters, the digital coding metamaterials are manipulated by simply designing different coding sequences to reach various EM functionalities. For instance, under the uniform coding sequence of “000000 … /000000 …”, the normally incident plane wave is mainly reflected to a single main beam at broadside, as shown in the left column of Figure 2C; under the periodic coding sequence of “010101 … /010101 …”, the normally incident plane wave is mainly reflected to two symmetrical beams, as shown in the middle column of Figure 2C, whereas in the case of periodic coding sequence of “010101 … /101010 … /010101 … /101010 …”, the normally incident plane wave is mainly reflected to four symmetrical beams, as shown in the right column of Figure 2C. Both theoretical and full-wave simulation results demonstrate that the digital coding metamaterials can manipulate the EM waves by simply using the pre-designed coding sequences. Due to the great flexibilities and simplicities of digital coding metamaterials, they have been extensively studied in the past five years for achieving many fruitful applications, such as reflect and transmit arrays, diffuse scattering, vortex beams generation, polarization control, holograms, computational imaging, and antenna designs.

### Programmable Metamaterials

Evolved from digital coding metamaterials, the first programmable metamaterial was presented in Ref. 55, with field programmable capabilities to realize diverse wave-manipulation functions. By pre-designing various coding sequences or coding patterns with the EM theory, coding theory, and other approaches, we can store the coding sequences and their corresponding functions into FPGA to realize the programmable metamaterial in real time. With the binary digits (0 and 1) and digital operations, the metamaterial configuration design and arrangement are significantly simplified, which significantly reduces the complexity of FPGA process. On the contrary, the conventional active metamaterials (like reconfigurable metamaterials and tunable metamaterials) have many more states and require remarkable calculation resources, restricting their application potentials in real-time reactions.

Here we introduce the first presentation of the programmable metamaterial (Cui et al., 2014) to illustrate its internal working mechanism. Based on the 1-bit digital coding atom in Figure 2B, a programmable metamaterial with 30◊30 digital atoms is designed, simulated, and measured. To clearly exhibit the performance, four coding sequences (000000, 111111, 010101, and 001011) are designed, in which each code represents a 5◊5 supercell. For the sequences 000000 and 111111, simulated and measured scattering patterns show that the reflected energy concentrates on the central beam, because they mimic the perfectly electric and magnetic conductors, respectively. For the coding sequence 010101, the incident energy is effectively reflected in two symmetry directions, whereas the sequence 001011 arranges the reflecting energy on the multiple directions, which achieves a lower radar cross section (RCS). To achieve the fast and programmable control, FPGA with four switches is equipped on the metamaterial to flexibly trigger the desired coding patterns, which are pre-stored in FPGA. As displayed in Figure 2D, when FPGA detects the trigger signal, the relevant bias-voltage will be implemented on each diode according to the coding sequences (000000, 111111, 010101, and 001011). Except for the above four coding sequences as the illustrative examples, we remark that much more coding patterns and functions can be designed using the coding metamaterial theory.

### Information Metamaterials

The programmable metamaterial not only simplifies the design can control the EM waves in real time but also has the feature to process the digital information. This important feature results in the appearance of information metamaterial (Cui et al., 2017). This subsection will introduce the concept and basic principles of information metamaterials.

#### Information Entropy

Entropy was initially introduced to describe the macroscopic behavior of a thermodynamic system, which consists of a huge number of atoms or molecules, and was later applied to the fields of quantum mechanics as von Neumann entropy and information theory as information entropy (also referred to as Shannon entropy). Shannon entropy, proposed by Claude Shannon in 1948, gave an upper limit for the attainable average length of lossless encoding (compression) of an information source (Shannon, 2001). A larger information entropy implies more uncertainty of the information source and thus the larger capacity of information it can carry.

**Figure 3:**
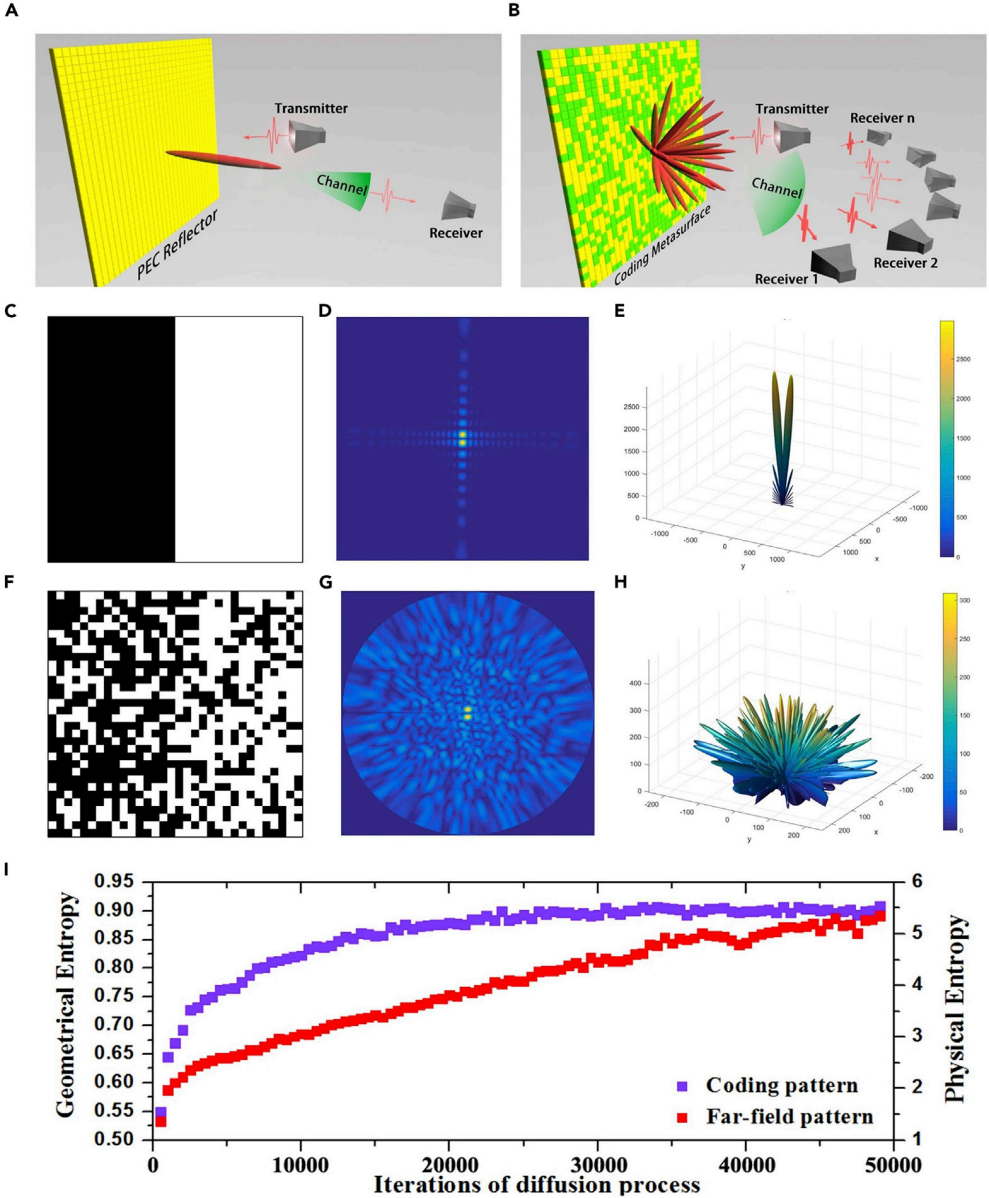
Information Entropy of the Digital Coding Metamaterial (A and B) Schematic illustration of the wireless communication system based on the programmable metamaterial. (C–E) Coding pattern and 2D/3D radiation patterns of the half 0 and a half 1 coding, respectively. (F–H) Coding pattern and 2D/3D radiation patterns of the random coding, respectively. (I) Geometrical entropy and physical entropy of the coding patterns generated in the diffusion process.

A programmable metamaterial can generate dynamically many different radiation patterns and thus can be considered as an information source where information is modulated in the far-field radiation patterns (Cui et al., 2016). Figures 3A and 3B sketch the conceived wireless communication system based on the programmable metamaterial, which mainly includes three parts: a transmitter, a receiver, and a channel. The transmitted signals passing through a wireless channel with multipath effect and noises interference are received by multiple receivers deployed at different positions in the far-field. An all-0-coding pattern functioning as a perfectly electric conductor reflects the incident beam to a single direction (Figure 3A), which contains less information. In sharp contrast, a random coding pattern diffuses the incident beam to multiple directions (Figure 3B) and thus potentially carrying a higher capacity of information. Thus it is of great importance if one could estimate the amount of information carried by a certain coding pattern.

Based on the definition of information entropy, we proposed, for the first time, a fast method to calculate the information entropy of a digital coding pattern using the following definition of information entropy (Cui et al., 2016)

(Equation 1) $$H_{2}=-\sum_{i=1}^{2}\sum_{j=1}^{2}P_{ij}\log_{\text{2}}P_{ij}$$

in which *P*_*ij*_ is the joint probability of a group *G*(*i, j*) representing two adjacent coding elements in the coding pattern. Note that the 2D information entropy reflects not only the proportion of the “1” and “0” elements in the coding pattern but also their spatial distributions. According to Equation (1), the appearance of four groups—*G*(0, 0), *G*(0, 1), *G*(1, 0), and *G*(1, 1) —in the coding pattern determines the 2D entropy.

To reveal the relationship between the entropy of coding pattern (geometrical entropy) and radiation pattern (physical entropy), we generate a series of random coding patterns using a cellular automata machine (Cui et al., 2016). The process starts with a half “1” and a half “0” coding pattern (see Figure 3C). As the random mixing process, the “1” and “0” coding particles gradually mix into each other, forming the random coding pattern shown in Figure 3F. Using fast Fourier transform (FFT), we calculate the 2D/3D radiation patterns and plot them in the polar coordinate (Figures 3D and 3G) and spherical coordinate (Figures 3E and 3H). As expected, the radiation pattern evolves from a single beam (Figure 3E) to the diffused pattern with multiple beams (Figure 3H). Figure 3I shows the evolution of the entropy of the coding pattern (geometrical entropy) and radiation pattern (physical entropy) in the entire process. Note that the radiation pattern is firstly transformed into the polar coordinate with grayscale coloring before it is ready for the calculation of physical entropy. The physical entropy is found to have an increasing trend with the geometrical entropy. It is interesting to note that this process mimics the mixing of two types of gas molecules in a container, where the system entropy experiences an increasing trend. With the proportional relation between the physical entropy and geometrical entropy, we could control the information of metamaterials by generating the coding patterns with desired geometrical entropy, which will have potential applications in the wireless communication systems.

A subsequent work further gives the upper bound of information contained in the radiation pattern of a digital coding metamaterial and reveals the theoretical upper limit of orthogonal radiation states that can be realized. It was found that the information-entropy derivative of the random coding pattern is equal to 1-*γ* (*γ* is the Euler's constant), which is irrelevant to the size, the number of elements, and the coding patterns (Wu et al., 2019). This work establishes a quantitative framework to characterize the information processing capabilities of coding metamaterials and provides guidance for the inverse design with desired functions.

#### Convolution Theorem

We know from the previous sections that the anomalous reflection/refraction angle of a coding metamaterial is determined by the periodicity of the gradient coding sequence. The attainable reflection/refraction angle is limited to certain discrete values for integer times of the smallest gradient coding sequence “0 1 2 3 0 1 2 3 …”, which seriously limits the application scope. By applying the convolution theorem from digital signal processing to the coding metamaterial, Liu et al., 2016dproposed a new coding strategy, called as the scattering pattern shift, which enables the rotation of a radiation pattern to an arbitrary direction with negligible distortion. This coding strategy allows us to generate many complicated radiation patterns that conventionally require brutal-force numerical simulations.

The principle of scattering pattern shift is inspired by the Fourier transform relation between the far-field radiation pattern and the coding pattern. Here, the basic principle of the convolution theorem is briefly reviewed as

(Equation 2) $$f\left(t\right)\cdot e^{j\omega_{0}t}\overset{FFT}{↔}f\left(\omega \right)*\delta \left(\omega -\omega_{0}\right)=f\left(\omega -\omega_{0}\right)$$

in which $e^{j\omega_{0}t}$ is the time-shift item in the time domain and the impulse function $\delta \left(\omega -\omega_{0}\right)$ is its frequency spectrum in the frequency domain. Equation (2) implies that the convolution of a spectrum f(ω) with an impulse function $\delta \left(\omega -\omega_{0}\right)$ results in a shift of the spectrum function f(ω) by a value of *ω*_*0*_ in the frequency domain without distortion. By replacing *t* and *ω* in Equation (2) with *x*_*λ*_ and sin *θ*, respectively, we obtain the function of the scattering pattern shift as (Liu et al., 2016a, Liu et al., 2016, 2016c, 2016d, 2016e)

(Equation 3) $$E\left(x_{\lambda }\right)\cdot e^{jx_{\lambda }\;\sin\;\theta_{0}}\overset{FFT}{↔}E\left(\sin\;\theta \right)*\delta \left(\sin\;\theta -\sin\;\theta_{0}\right)=E\left(\sin\;\theta -\sin\;\theta_{0}\right)$$

where the item $e^{jx_{\lambda }\;\sin\;\theta_{0}}$ represents a coding pattern with a gradient phase along a certain direction. Equation (3) describes the equivalence between the multiplication of a coding pattern *E(x*_*λ*_*)* with a gradient coding pattern $e^{jx_{\lambda }\;\sin\;\theta_{0}}$ and the convolution of their radiation patterns. The resulting radiation pattern $E\left(\sin\;\theta -\sin\;\theta_{0}\right)$ is diverted to the direction of the gradient coding pattern while inheriting most features of the original coding pattern.

**Figure 4:**
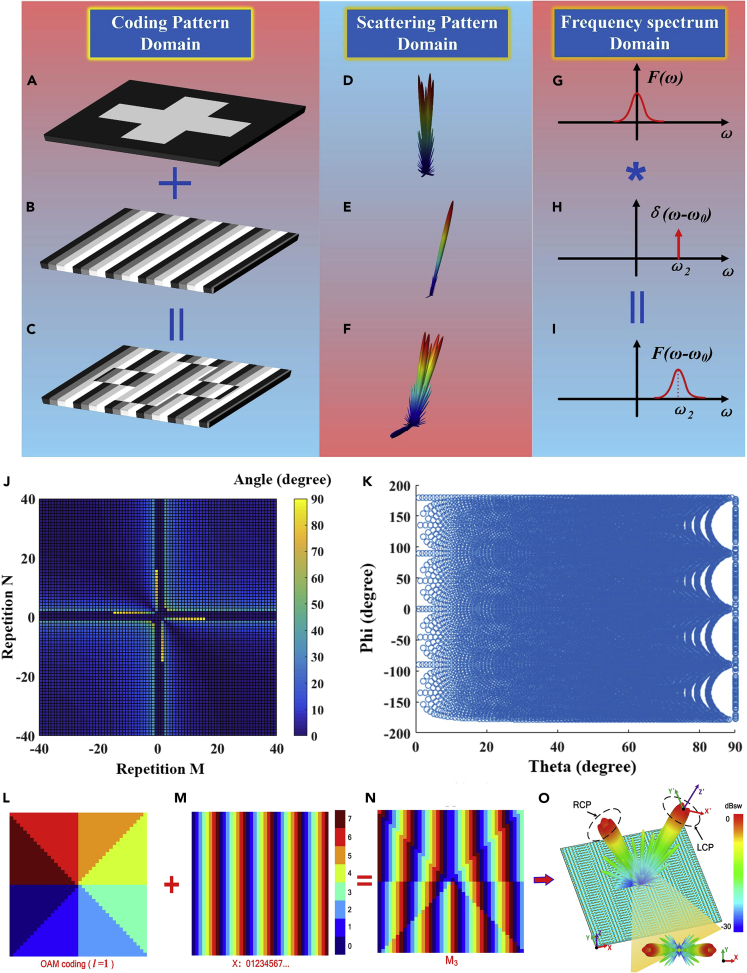
Principle of Scattering Pattern Shift and Its Applications (A–C) The coding pattern domain. (D–F) The calculated radiation patterns corresponding to the coding patterns in (A–C), respectively. (G–I) The equivalent frequency spectra of the radiation patterns in (D–F), respectively. (J) The attainable radiation angles calculated by adding two gradient coding sequences with different super-unit-cell sizes *M* and *N*. (K) The attainable angle of the mixed coding pattern when the two gradient coding sequences vary along with orthogonal directions. (L) The coding pattern for the OAM mode *l* = 1. (M) The coding pattern with gradient coding sequence “0 1 2 3 4 5 6 7···” varying along the *x* direction. (N) The mixed coding pattern of (L) and (m). (O) The radiation pattern of the mixed coding pattern.

The principle of the scattering pattern shift is schematically illustrated in Figures 4A–4I. The five-beam radiation pattern generated by a cross-shaped coding pattern (see Figures 4A and 4D) will be rotated to an anomalous direction (see Figures 4C and 4F) by making convolution operation with a single-beam radiation pattern (see Figures 4B and 4E) (Liu et al., 2016a, Liu et al., 2016, 2016c, 2016d, 2016e). The convolution of two coding patterns is simply the modulus of their coding digits. Interestingly, this process mimics the frequency-shift theorem, where the original frequency spectrum (see Figure 4G) is shifted to a higher frequency *ω*_*2*_ (see Figure 4I) by making convolution operation with Dirac delta function as shown in Figure 4H.

Using the convolution theorem, we can principally generate the beam radiations at arbitrary angles with 2-bit digital coding, thus producing continuous scanning ability of radiation beams (Liu et al., 2016a, Liu et al., 2016, 2016c, 2016d, 2016e), as illustrated in Figures 4J and 4K. For example, Figure 4J shows the attainable radiation angles calculated from the addition of two gradient coding sequences with different super-unit-cell sizes *M* and *N* (i.e., the repetition numbers of each digit in the coding sequence), which covers almost all values from 0° to 90° in the elevation plane. The beam can also sweep in the azimuthal plane by adding a gradient coding sequence that varies along the orthogonal direction, as shown in Figure 4K, where each circle represents an attainable radiation direction in the entire upper-half sphere (Liu et al., 2016a, Liu et al., 2016, 2016c, 2016d, 2016e).

The digital convolution operations on a Pancharatnam-Berry (PB) coding metamaterial can also generate spin-controlled vortex beams carrying orbital angular momentum with different polarizations (Zhang et al., 2017). Figures 4L–4O illustrate the results of PB coding metamaterials that generate vortex beams with OAM mode *l* = 1. The coding pattern in Figure 4L consists of the gradient coding pattern in the azimuthal direction, which generates a vortex beam pointing in the normal direction. By adding it with a gradient coding pattern along the *x* direction (see Figure 4M), we obtain a combined coding pattern in Figure 4N, which generates the tilted vortex beams pointing in the directions of *θ* = ±24.6°.

#### Addition Theorem

In parallel with the convolution theorem, another coding scheme called as addition theorem was proposed (Wu et al., 2018), which allows independent controls of multiple radiation beams. We notice the fact that the phase response of a unit cell is expressed in the complex form $e^{j\phi }$ instead of itself ϕ. The complex code contains the full phase information of EM waves and possesses a higher degree of freedom in the coding pattern design to generate more flexible radiation patterns.

**Figure 5:**
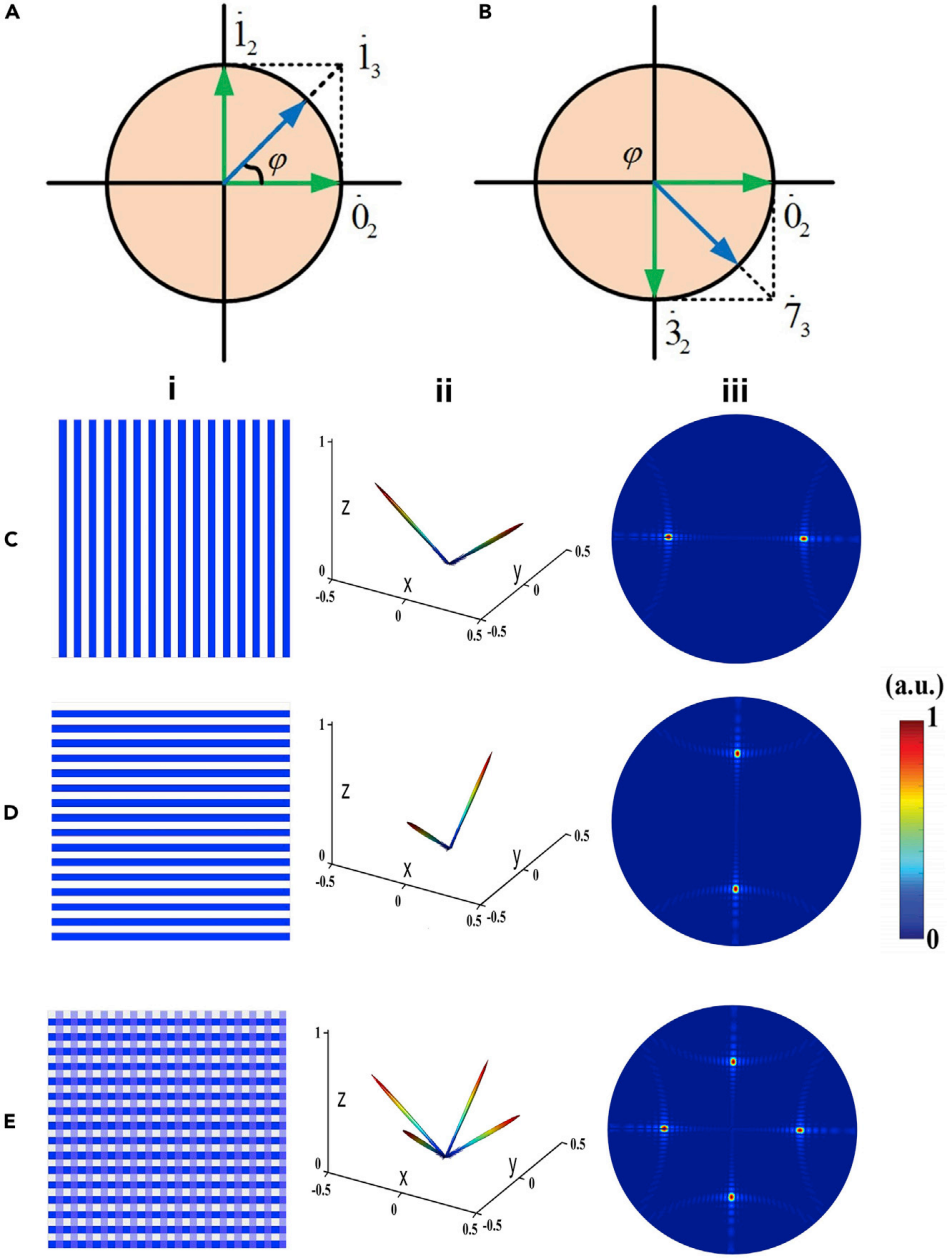
Addition Theroem to Allow More Complicated Radiation Patterns (A and B) Two typical addition processes of 2-bit complex codes for ${\dot{0}}_{2}+{\dot{1}}_{2}={\dot{1}}_{3}$ (A) and ${\dot{0}}_{2}+{\dot{3}}_{2}={\dot{7}}_{3}$ (B) on the coding circle. (C–E) Coding schemes and their 3D and 2D scattering patterns calculated by FFT to illustrate the influence of indefinite coding addition. (C) The coding sequence 00110011··· along the *x*-direction. (D) The coding sequence 00110011··· along the *y*-direction. (E) The addition of the coding patterns in (C) and (D) using the new coding scheme by correcting the indefinite coding elements. (i) Coding patterns. (ii) 3D radiation patterns. (iii) 2D radiation patterns.

Before we introduce the addition theorem for a complex coding scheme, we should firstly give the definition of complex digital codes. As shown in Figures 5A and 5B, all complex digital codes are located on a unit circle, named as “coding circle,” on which arbitrary-bit digital states can be denoted on the coding circle by unit vectors. The addition operation of two complex digital codes is

(Equation 4) $$e^{j\phi_{1}}+e^{j\phi_{2}}=Ae^{j\phi_{0}}$$

which produces a new complex number with phase $\phi_{0}$ and magnitude A. In fact, the addition operation can be realized by vector superposition principle in the traditional Euclidean geometry. For example, the addition of two 2-bit complex-codes ${\dot{0}}_{2}+{\dot{1}}_{2}$ gives a phase of 45°, which corresponds to the complex digital state ${\dot{1}}_{3}$in the 3-bit coding set, as illustrated in Figure 8A, whereas ${\dot{0}}_{2}+{\dot{3}}_{2}$ results in a phase of 315°, corresponding to the complex digital state ${\dot{7}}_{3}$ in the 3-bit coding, as illustrated in Figure 5B. The above example shows that the addition of two *N*-bit complex codes produces an (*N*+1)-bit complex code. This implies that we can obtain any higher-bit complex coding sets by iterating the addition operations several times from the simplest 1-bit complex codes (Wu et al., 2018). However, there is a situation when the addition of two complex codes having opposite phases results in zero value, which is called as “indefinite coding addition” because the phase of zero is indefinite. This issue should be solved before we set rules for the addition operations. Theoretical analysis shows that the indefinite-element rate of N-bit complex codes is $2^{N}/2^{2N}=1/2^{N}$ for any coding pattern. Thus, 1-bit complex codes suffer the biggest indefinite-element rate of 0.5 and can become even larger if the probability of every code is different in some special cases.

An example is given in Figures 5C–5E to illustrate the performance of the addition theorem in combining two dual-beam radiation patterns (Figures 5C and 5D) into one quad-beam radiation pattern (Figure 5E) (Wu et al., 2018). However, the direct addition of the dual-beam coding patterns in Figure 5CI and 5DI results in obvious radiation in the 0° direction, due to the missing of digital states resulted from the massive indefinite elements in the additive coding pattern. To fix this issue, a set of regulations are established to break the consistency of identical code distribution on the aperture caused by indefinite additions, while keeping the rationality. The performance of the new addition rules is validated in Figure 5E, in which the incident beam is equally deflected to four pencil beams without observable back scatterings.

## Information Metamaterial Systems

From the discussions in the earlier section, the information metamaterial has three main features: (1) it can realize many different functions in a programmable way; (2) it can manipulate the EM waves in real time with the help of FPGA; and (3) it can control and process the EM waves and digital information simultaneously. These features make the information metamaterial no longer an effective material, but an information system. In this section, we will introduce the system-level applications of information metamaterials and present four typical types of systems in details: field programmable systems, software metamaterial systems, intelligent metamaterial systems, and space-time-coding digital metamaterial systems.

### Field Programmable Systems

Field programmable systems are direct applications of the programmable metamaterials. Based on the functions to be realized and the ways to control the digital switches, below we present several field programmable systems.

#### Programmable Radar

In electronic scanning radar systems, active phased arrays are crucial parts for beam steering. Although active phased arrays have long been served in military radar systems, they are difficult to be used directly in commercial applications. Because they need massive transceivers to realize beam steering, the whole system will be very complicated and expensive. The programmable metamaterial enables direct modulations of EM waves by designing the coding sequences, thus providing a new way to realize beam steering. Here, we present a direct digital mechanism to control the scattered EM waves based on the programmable metamaterial, in which each element loads a PIN diode to produce the binary states of “1” and “0” (Cui et al., 2014; Wan et al., 2016). Through data cables, all the binary states of the programmable metamaterial are controlled by FPGA. The new mechanism has great potentials in new-concept radars and communication systems.

**Figure 6:**
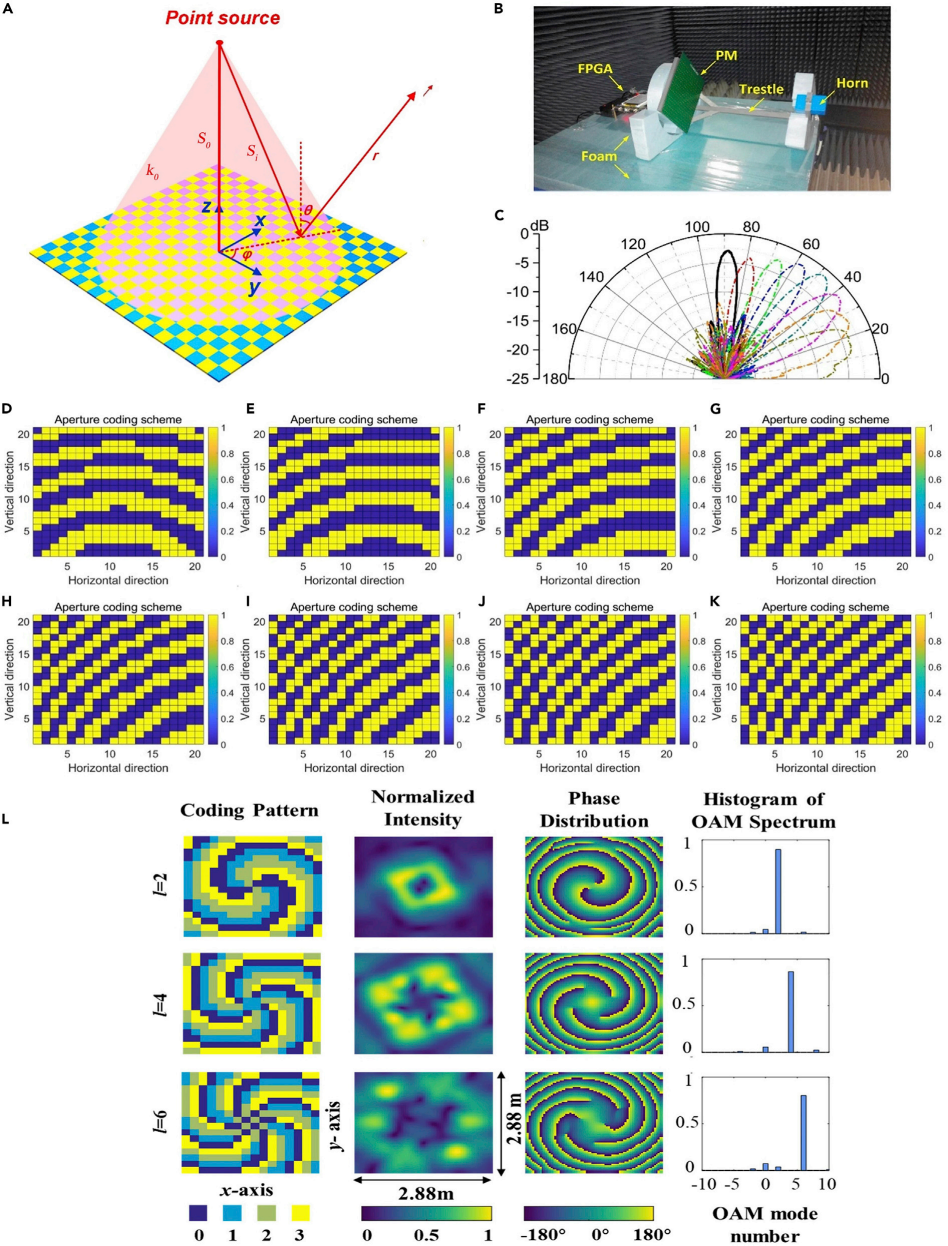
Programmable Radar and Programmable Vortex-Beam Generator (A) The point-source excited programmable metamaterial. (B) Photograph of the programmable radar. (C) Measured results of beam steering. (D–K) The coding schemes corresponding to the scanning angles from 0° to 70° with an increment of 10°. (L) Experimental results of single-mode OAM-carrying beams with different topological charges of l = 2, 4, and 6.

A programmable metamaterial consisting of 400 (20×20) elements is proposed to realize beam steering at the central frequency of 8.9 GHz by designing the coding schemes (Wan et al., 2016). As illustrated in Figure 6A, a point source (such as the feeding horn antenna) instead of plane wave is used in the radar system. Then the phase coding on the metamaterial must be compensated first. Supposing that a directional beam in a specific direction is produced by the programmable metamaterial, the reflection phase of each coding element should satisfy the following equation

(Equation 5) $$\varphi_{i}\left(\theta ,\;\phi \right)=k_{o}\left(S_{i}-S_{o}-x_{i}sin\theta cos\phi -y_{i}sin\theta sin\phi \right)+\varphi_{o}$$

where $\;k_{o}\;$ is the wave number in free space; $S_{o}\;$ is the shortest distance between the source and metasurface, and $\;S_{i}\;$ is the distance between the coding element *i* and the source; $\varphi_{i}\;$ is reflective phase of the element, and $\;\varphi_{o}\;$ is an arbitrary initial phase; and θ  and  ϕ  are elevation and azimuth angles of the sum beam, respectively. Figures 6D–6K give the reflection phase distributions of the programmable metamaterial when the elevation angle of directional beam steers from 0° to 70°. Because the elements of the programmable metamaterial exhibit digital states, the reflection phases have to be digitized. Specifically, if $\;\phi_{i}\in (0,\pi ]$, the element is represented by blue patch, otherwise, by yellow patch. Thus, the phase distribution is digitalized to coding schemes of the programmable metamaterial. During design and implementation, the blue patches denote off-state PIN diodes, and the yellow patches denote on-state PIN diodes.

The fabricated programmable radar is shown in Figure 6B, in which a horn antenna is used to illuminate the programmable metamaterial, and a plastic trestle is designed to fix the horn antenna and the metamaterial. We have designed FPGA to control the digital coding schemes in real time, so that the directional beam can be steered to specified directions. The digital coding schemes corresponding to different directions are stored in FPGA in advance, so that the correct digital coding scheme is recalled during each measurement. The whole system is placed on a platform of microwave anechoic chamber, as can be seen from Figure 6B. During measurements, the programmable metamaterial served as the transmitter when the platform rotates. A receiving horn antenna is used to record the signals and measure the far field patterns. Figure 6C gives the measured far-field patterns corresponding to the coding schemes in Figures 6D–6K. As expected, the elevation angle of the directional beam steers from 0° to 70°.

#### Programmable Vortex-Beam Generator

In the past decades, the EM waves carrying OAM have gained intensive interests, because they could potentially provide additional degrees of freedom of EM information. Although various approaches to generating OAM beams have been developed, they are hardly deployed in a real-world scenario because they cannot be dynamically generated in real time and low cost, especially for the case of topological charges more than 2. To resolve this problem, an inexpensive 2-bit coding programmable metamaterial was explored for generating high-order OAM-carrying beams in a programmable way. Based on the designed metamaterial, we experimentally realized the generation of OAM beams with the electronically controlled vortex centers and topological charges of ℓ = 0, ±1, ±2, ±3, ±4, ±5, and ±6. The designed 2-bit programmable metamaterial could find its valuable applications in direct-modulation wireless communications (Shuang et al., 2020).

Figure 6L reports the experimental results to demonstrate the origin-centered single-mode OAM-carrying beams with different topological charges—ℓ=2,  4 ,  6—when the metamaterial is programmed with different coding patterns (Shuang et al., 2020). In the first column, the coding patterns of the metamaterial for generating the origin-centered single-mode OAM-carrying beams are plotted. The corresponding amplitudes and phases are presented respectively in the second and third columns. The spectrum decomposition of OAM is further performed, and the results are shown in the rightest column of Figure 6L. The same programmable metamaterial can also be used to produce multi-mode OAM-carrying beams with controllable vortex centers. For example, we have realized dual-mode OAM beams with the topological charges *l* = −1 and *l* = 1 to steer to θ = 30° and θ = 20° in the *yoz* plane, respectively and triple-mode and four-mode OAM-carrying beams with different topological charges and vortex centers in both *yoz* and *xoz* planes (Shuang et al., 2020). This strategy will bring a fundamental perspective on designing new wireless communication architectures at various frequencies.

#### Multichannel Data Transmission System

By configuring the digital coding elements, a programmable metamaterial can construct dynamic near-field imaging patterns. Because the aperture codes of the programmable metamaterial change over time, the digital coding mechanism naturally involves temporal operations. Hence, for each position on the near-field plane, the amplitudes and phases of the near fields can be modulated in the time domain by sequentially changing the aperture codes. Therefore, the programmable metamaterial can perform near-field modulations in both space and time domains, which can be used to directly transmit near-field information in multiple channels (Wan et al., 2019). As an example, three points are selected in the near-field region to form three independent channels. By applying various digital phase codes on the programmable metamaterial, three independent binary digital symbols defined by amplitude codes (weak and strong amplitudes are 0 and 1 amplitude codes) are transmitted through the three channels. The measured near-field distributions and temporal transmissions of the system agree with numerical calculations. As the spatial and temporal modulations are directly realized by the programmable metamaterial, the presented mechanism will enrich the modes of 5G wireless communication and improve the mechanisms of near-field information processing and communications.

**Figure 7:**
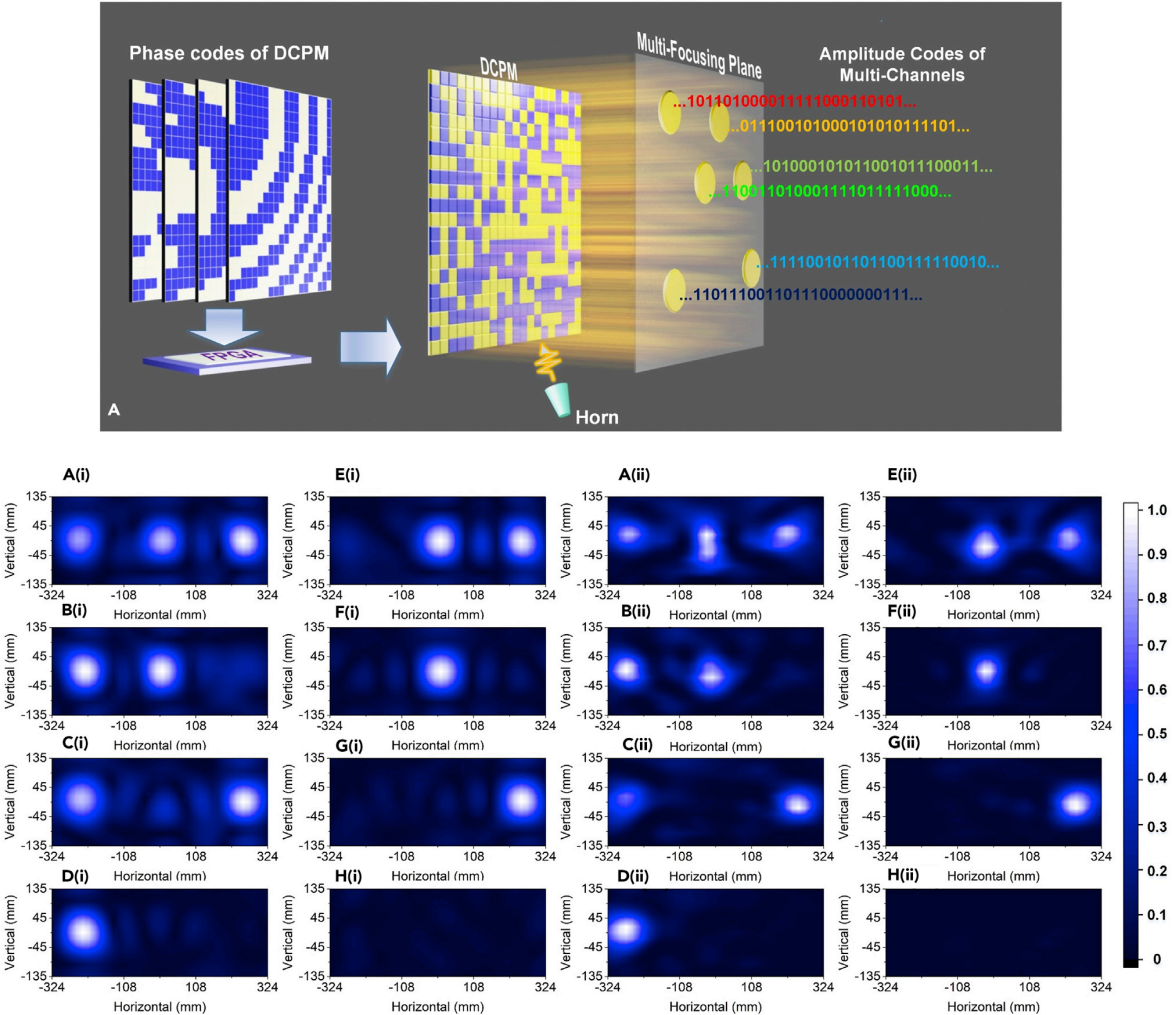
Diagram of the Multichannel Information Transmission System and Near-Field Patterns of Various Combinations of Symbols in Three Channels (A) The coding schemes are stored in FPGA in advance and are recalled to sequentially configure the programmable metamaterial so that the near fields are modulated in the space and time domains. As a result, distinct information can be transmitted through each channel. A(i) to H(i) The simulated results. A(ii) to H(ii) The measured results.

A conceptual diagram of the multichannel transmission system is presented in Figure 7A, in which the programmable metamaterial consists of 400 elements (Wan et al., 2019). Each element can render either of the digital states possessing opposite reflection phases and similar reflection amplitudes. The aperture configurations of the programmable metamaterial can be described by a matrix of the binary phase codes. By applying specially designed phase codes, the digital metamaterial can focus the EM fields at arbitrary specified points and the field intensities at these points can be controlled independently. If one denotes strong field intensity as digital symbol “1” and weak field intensity as digital symbol “0” in amplitude coding, these points will function as multiple transmission channels of binary symbols. To continuously transmit the binary digital symbols, the programmable metamaterial is configured sequentially via a series of coding schemes, which are stored in FPGA in advance. As a result, different binary symbols can be independently transmitted at different near-field points, thereby realizing multichannel direct transmissions of the near-field information. The near-field patterns of the system are presented in the lower part of Figure 7, in which the left two columns are calculated results and the right two columns are measured results. We observe that the intensities of three points are modulated independently by changing the coding schemes of the programmable metamaterial. The three points form three transmission channels, and binary digital symbols can be transmitted through these channels. Comparing with the conventional multichannel transmissions, the new transmission mechanism does not require any phase shifters for spatial modulations or baseband modulators for temporal modulations. These advantages endow the programmable metamaterial with high potentials in the near-field information processing and high-capacity communications.

#### Light-Controlled Programmable Metamaterial System

In abovementioned programmable metamaterials, the digital meta-atoms are controlled by the PIN diodes, which must be physically connected to the power suppliers by electrical wires. This issue will cause technical problems when large amounts of meta-atoms are needed. To solve the problems, a remote-mode and wireless programmable metamaterial controlled by illuminating light was proposed (Zhang et al., 2018a, 2018b). The presented light-controlled programmable metamaterial is composed of digital meta-atoms and an array of photodiodes, which provides light-controllable DC bias for the varactor diodes embedded in the digital meta-atoms. To generate the expected scattering or radiating waves, the corresponding coding sequences are firstly predesigned and then the signal is sent by light source array. After that, the photodiode array will receive the coding sequence and transmit it to the metamaterial. Finally, the incident waves will be dynamically controlled by the digital coding metamaterial. Hence, the waves can be controlled by the “coded” lights emitted by the source array.

**Figure 8:**
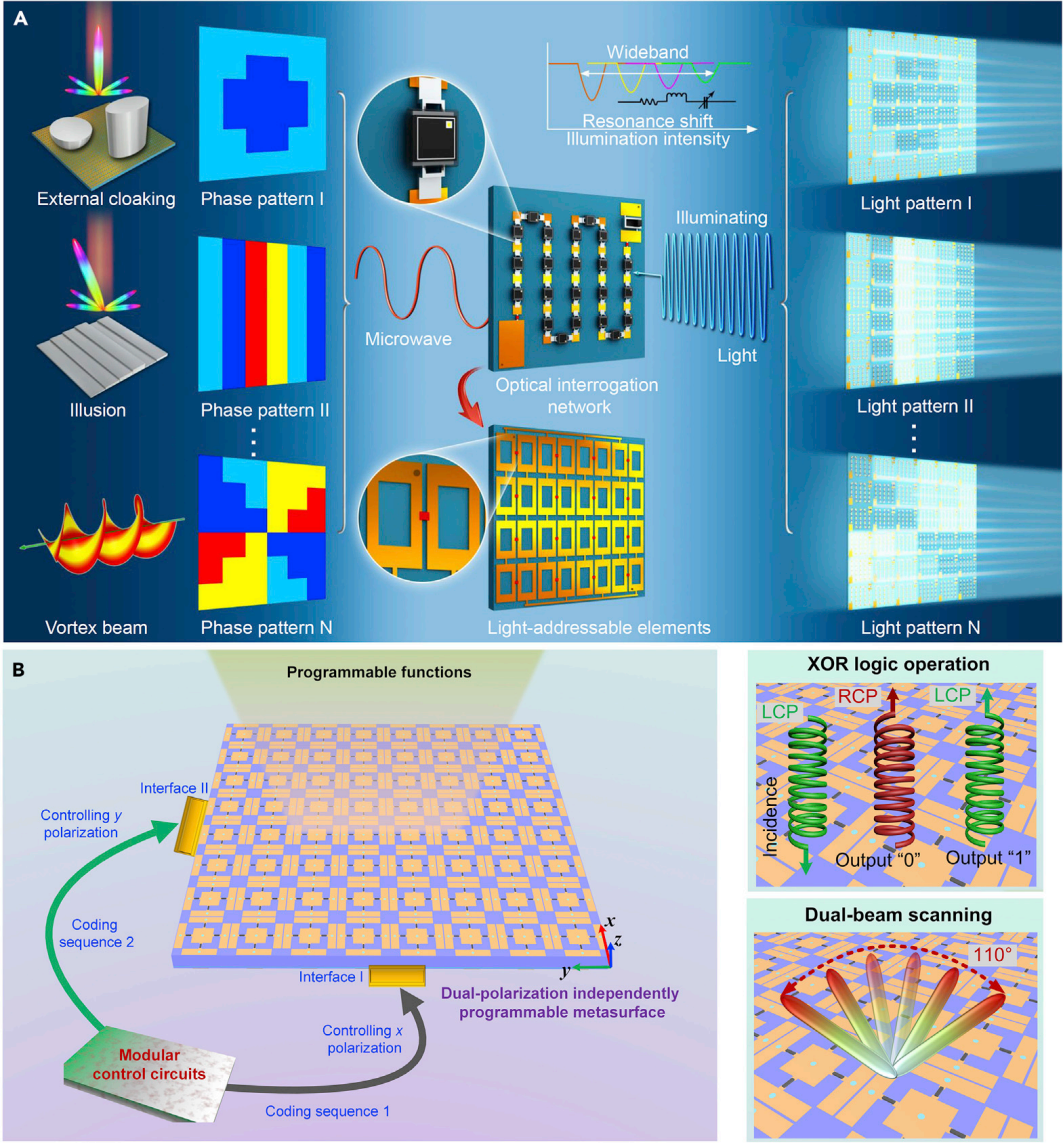
Optically Interrogated Digital Metamaterial (OIDM) to Realize Light-Controlled Programmable Metamaterial System (A) Illustration of OIDM and its programmable functions. For different light patterns, OIDM can realize different functions, such as external cloaking, microwave illusion, and dynamic vortex generation. (B) Illustration of PDPM for independent control of orthogonal-polarized waves. The realized system can achieve versatile functions, such as wave-based XOR logic operation for spin control, wide-angle dual-beam scanning, and dual-polarized aperture sharing.

Recently, the advanced version, an optically interrogated digital metamaterial (OIDM) with more rich programmability has been proposed and experimentally realized (Zhang et al., 2020a). The constructed programmable metamaterial works in wideband and can be switched by multiple independent optical interrogation networks (OINs). On receiving the light patterns, OIN acts as both DC power and controller. The overall thickness of the presented OIDM is only approximately 0.085 wavelengths at the working frequency band. The principle and structure of OIDM is illustrated in Figure 8A. Here, OIDM consists of 6 × 6 subarrays, each of which contains 4 × 4 elaborately designed light-addressable meta-atoms and an OIN integrated on the back of the meta-atoms. When the “coded” light is projected onto OIN, different illumination intensities will be converted to different voltages that are applied to the corresponding meta-atoms, and their resonant states are switched remotely. Each subarray can be controlled independently, thus the programmable OIDM can generate various reflection phase distributions for different light patterns and realize different functions.

The fabricated OIDM contains metallic artificially designed structures on the top and metal ground on the bottom (Zhang et al., 2020a). To achieve tunable effective capacitance, a varactor is loaded in the center of the gap between two designed rectangular rings. To drive the varactor effectively, each rectangular ring has two stubs at its ends, serving as bias lines. To realize the low-Q tunable element in wide-frequency band, a suitable varactor with low ohmic loss and high capacitance ratio was chosen. OIN contains a series of photodiode arrays that receives different optical signals and converts them into the corresponding voltage signals for tuning the loaded varactor. The OIN layer and super unit cells are connected to form an optically interrogated subarray. Hence, the subarray can control EM waves dynamically by receiving the light with different illumination intensities. The fabricated OIDM contains 6 × 6 such subarrays and achieves rich programmable capabilities. As a proof of concept, the functions of external cloaking, illusion, and dynamic vortex-beam generation are demonstrated distinctly based on the single digital platform (Zhang et al., 2020a).

#### Dual-Programmable Metamaterial System

The programmable ability of the digital coding metamaterials can be further extended. Here, we introduce a polarization-controlled dual-programmable metamaterial (PDPM) for independent manipulations of orthogonally polarized waves (Zhang et al., 2020b). Inside PDPM, there are two sets of control interfaces, which are used to receive two independent coding sequences from FPGA, as shown in Figure 8B. The *x*- and *y*-polarized waves can be controlled independently by the two coding sequences. Hence, for the programmable metamaterial, the meta-atoms are able to manipulate two orthogonally polarized waves individually. The designed unit structure is composed of one square patch in the center and four surrounding metallic strips. For such a metallic pattern configuration, there are four gaps: the two gaps in the *x*-direction can provide the equivalent capacitances of the resonator for the *x*-polarized incidences. The case of other two gaps in the *y*-direction is similar. To achieve more tunable capacitance, four identical varactors are integrated in four gaps. When the *x*- and *y*-polarized incident waves are projected onto the designed metamaterial individually, the meta-atom will generate different phase profiles for the orthogonal polarization states. A DC bias network is designed to provide reverse bias voltages for the two pairs of varactors independently. The two metallic strips in the *x*-direction are connected to the bias line I through two metallic vias, and the other two metallic strips in the *y*-direction are connected to the bias line II by the other two metallic vias.

When the *x*- or *y*-polarized wave propagates normally into the designed meta-atom, changes of the capacitance of the two varactors in the cross-polarization direction have no effect on the resonance response. Hence, the designed meta-atom has high polarization stability and cross-polarization isolation. When the capacitance values change, the reflection amplitudes of the meta-atom can reach above −1 dB at 5.85 GHz and are larger than −2 dB in the whole working frequency band. Based on the proposed meta-atom, a 1-bit PDPM is designed and used to control two orthogonally linearly polarized EM waves in real time (Zhang et al., 2020b). The fabricated PDPM is composed of periodically arranged meta-atoms and contains two sets of control interfaces I and II, as shown in Figure 8B. The two interfaces are connected to control circuits independently. The incident *x*- and *y*-polarized waves can be programmed simultaneously with two different coding sequences through the two sets of control interfaces. Three advanced electronic systems were demonstrated using the fabricated PDPM prototype: a wave-based dynamic XOR logic gate platform, a dual-beam scanning antenna system, and a dual-polarized shared-aperture antenna system. Hence, the presented PDPM has more degrees of freedom to enrich the EM functions of the programmable metamaterials and increase the capability of digital information.

### Software Metamaterial Systems

In the programmable metamaterials, we usually predesign the digital coding patterns for different functions and store them in FPGA to realize the real-time controls. When designing the digital coding patterns, some algorithms or software may be used to fulfill some specific functions, such as imaging. Such algorithms and software can also be cooperated with FPGA, resulting in the software metamaterials. Below we introduce two typical software metamaterial systems.

#### Single-Sensor Single-Frequency Imaging

In the development of microwave imaging techniques, single-sensor imager has attracted great attention due to its excellent performance. A coded aperture with a series of random masks could provide enough information for a single sensor to collect. By solving an optimization problem, the original information from the probe can be retrieved with reduced number of measurements. Compared with the conventional imaging techniques, the coded aperture with a single sensor can effectively reduce the measurement quantity. But the main challenge of this architecture is the controllable mask, which can be easily overcome by the programmable metamaterial. In fact, the programmable metamaterial with voltage-controlled aperture can easily obtain the sufficient distinct scattering patterns to reconstruct the object information. The coding patterns composed of digits 0 and 1 provide flexible and fast modulations on these mask patterns, further facilitating the processing speed. Also, the controllable and alterable mask under single frequency could avoid the object dispersion, which happens when the transforming frequency-dependent masks are used in dispersive and resonant metamaterials (Lipworth et al., 2013; Hunt et al., 2013). Here, we introduce two representative works of single-sensor single-frequency imaging using the programmable metamaterials (Li et al., 2016; Wang et al., 2016).

**Figure 9:**
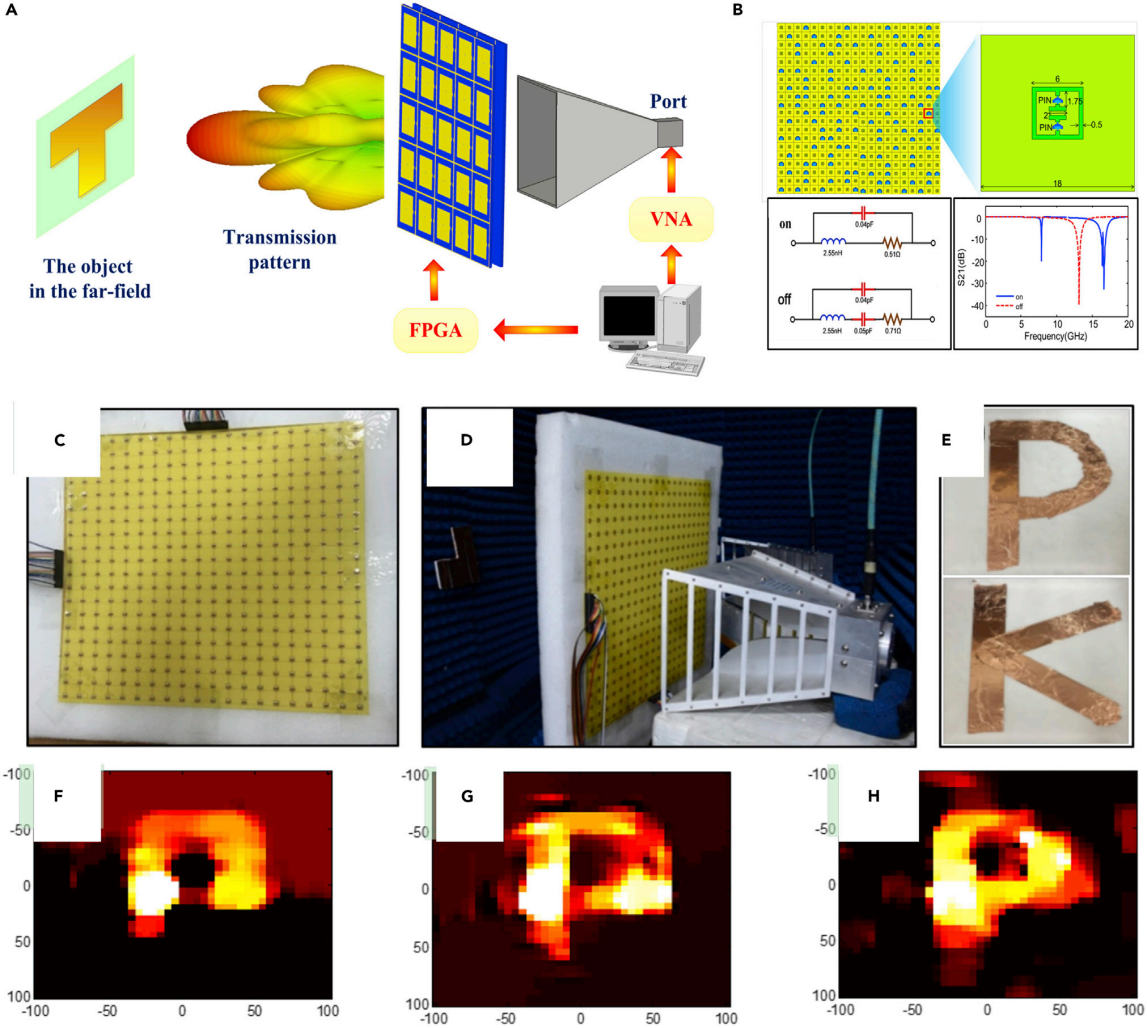
Transmission-Type 2-Bit Programmable Metasurface for Single-Sensor and Single-Frequency Microwave Imaging (A) A programmable metamaterial for single-sensor single-frequency imaging. (B) The metamaterial configuration and the meta-atom design. (C) The fabricated metamaterial. (D) The measurement arrangement. (E) The target objects. (F–H) The reconstructed images under 200, 400, and 600 measurements.

Figure 9A presents a single-sensor single-frequency imaging system using a transmissive programmable metamaterial (Li et al., 2016). Two PIN diodes are employed in each meta-atom to attain 2-bit phase modulation. A digitally controllable mask is set in front of a rectangular horn antenna, connecting to FPGA for fast pattern switch. In each pattern switch, the computer instructs the vector network analyzer to collect data once. After many times of measurements at the same frequency, the coefficient matrix is established through the Green's function as

$$\left[\begin{array}{c} V^{\left(1\right)}\\ V^{\left(2\right)}\\ \vdots \\ V^{\left(P\right)} \end{array}\right]=\left[\begin{array}{cccc} G_{11} & G_{12} & \ldots & G_{1N}\\ G_{21} & G_{22} & & \\ \vdots & & \ddots & \\ G_{P1} & & & G_{PN} \end{array}\right]\left[\begin{array}{c} \sigma_{1}\\ \sigma_{2}\\ \vdots \\ \sigma_{N} \end{array}\right]$$

where σ, G, and V are object-area vector, system response matrix, and measurement results, respectively, and *P* and *N* are the numbers of image pixels and measurement data. After solving the above matrix equation, the image of the target object will be obtained. However, in this Green's function-based inverse algorithm, the number of imaging pixels is very limited (Li et al., 2016).

To solve the problem, we further introduced the advanced signal processing algorithm, the compressed sensing (CS), in the single-sensor and single-frequency imaging system (Wang et al., 2016; Li et al., 2018). In Figure 9B, a 1-bit coding programmable metamaterial with larger aperture and more meta-atoms is presented to achieve the imaging purpose at a single frequency. Here, the detailed structure of the meta-atom embedded with two PIN diodes is given, in which the varied circuit models of diodes induce distinct transmission characteristics to encode the digital states as 0 and 1. Because the two diodes in each meta-atom share the same bias voltage, each meta-atom produces 1-bit transmission responses. In order to match with the CS algorithm, the whole metamaterial uses a column-row-wised manner to activate the related meta-atom, which prominently simplifies the control complexity. Therefore, the coding state of meta-atom is determined by the related voltage difference implemented by the column and row bias line.

Usually, it is best to use as few as possible of sequential measurement numbers in the imaging algorithm, so that the shutter control mechanism of the coded exposure can be much simpler. Traditionally, the coding pixels of the random mask are controlled independently, which limits the temporal and spatial resolutions. To break the bottleneck, we propose the column-row-wised coding metamaterial, by which the temporal random modulation of an $N_{x}\times N_{y}$ pixel array could be controlled by $N_{x}+N_{y}$ rather than $N_{x}\times N_{y}$ random binary sequences (Li et al., 2018). Here, $N_{x}$ and $N_{y}$ are the pixel numbers in the row and column directions, respectively. The row and column binary control signals jointly produce the binary random coded exposure sequence at the pixel location of ($n_{x},n_{y}$). Thus, the CS algorithm could drastically reduce the complexity and increase the efficiency.

A 20◊20 element metamaterial sample was fabricated, as shown in Figure 9C. By applying the pre-designed coding sequences such as 10111110001010100010 and 00110001110110001010 on the column and row control cables, we totally achieved 1,000 transmission patterns as database to reconstruct the images. Two rectangular horn antennas are applied as transmitter and receiver respectively, as depicted in Figure 9D. Two objects, the pasted copper letters “P” and “K,” are fabricated as the reconstruction targets (see Figure 9E). The measured results for the object “P” are presented in Figures 9F–9H, in which different measurement numbers are applied as 200, 400, and 600, respectively (Li et al., 2018). We clearly observe that the shape of the object letter “P” can be distinguished, validating good performance of the CS-based single-sensor and single-frequency microwave imaging.

#### Programmable Holographic Imaging

**Figure 10:**
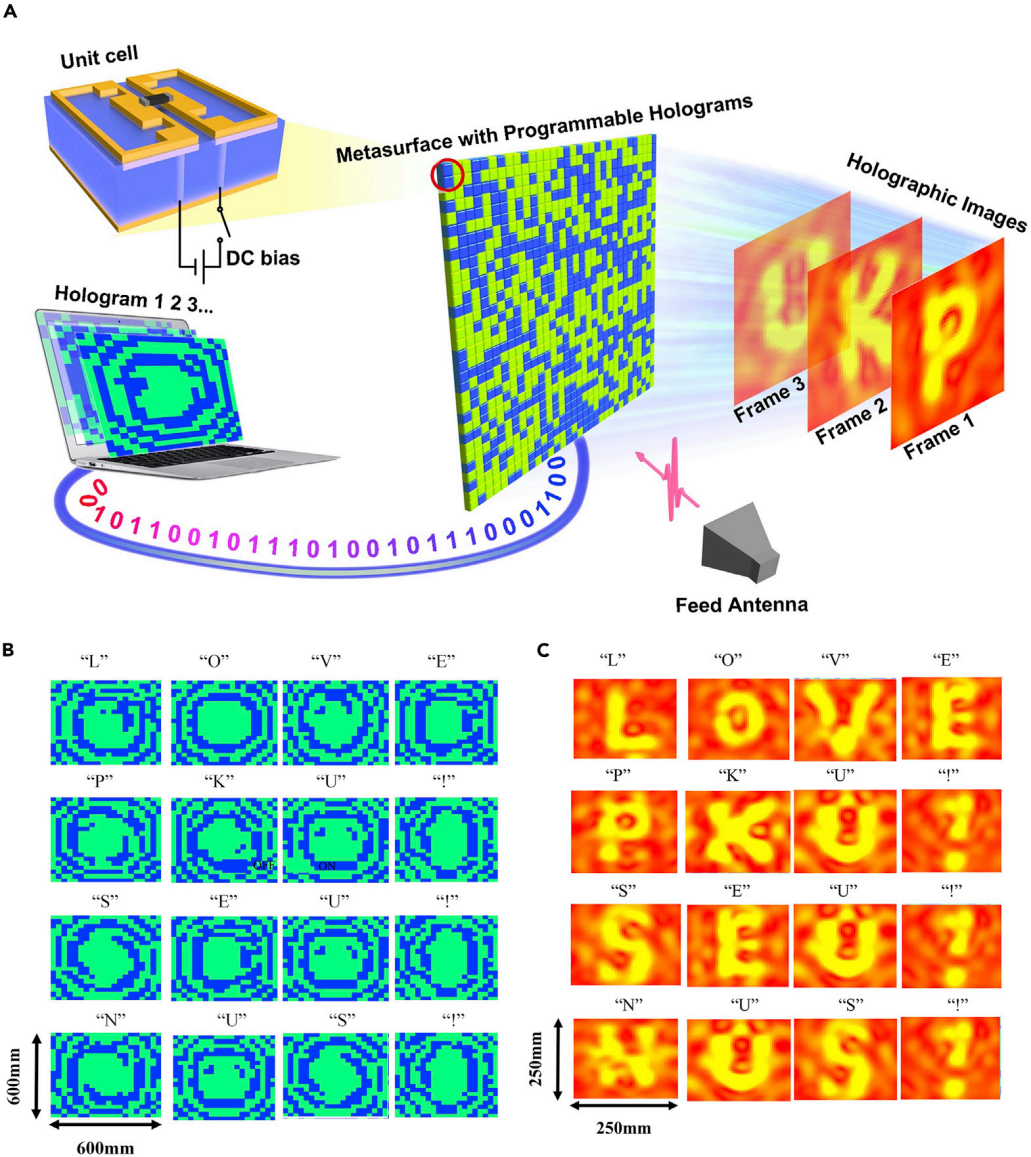
Programmable Holographic Imaging Based on 1-Bit Programmable Metamaterial (A) A sketch of the programmable holographic imaging. (B) The calculated digital coding patterns of the target images based on the modified GS algorithm. (C) The experimentally observed holographic images of “LOVE PKU! SEU! NUS!” in programmable way. Reference: L. Li, NC (2017)

By now, although a number of metamaterial holograms have been proposed in various frequency regimes to achieve holographic images with high efficiency, good image quality, and full colors, they are limited to the “static” scenario in the sense that only one or a few specific images can be generated once the metamaterial is fabricated. To beat this limitation, we presented the first programmable hologram with high-rate-of-frame (up to gigahertz) by designing a 1-bit coding programmable metamaterial (Li et al., 2017). This work addressed several critical issues typically associated with the current static metamaterial holograms, featuring the simplicity, being rewritable, high image quality, and high efficiency. Figure 10A illustrates the sketch map of the programmable holograph imaging based on the programmable metamaterial with 20 × 20 macro meta-atoms (Li et al., 2017), where the 1-bit meta-atom is plotted as well. For each meta-atom, two planar metallic structures are printed on top of substrate, with a PIN diode loaded between them. By incorporating the PIN diode into the meta-atom, the EM state of meta-atom can be electronically controlled by applying different biased voltages across the diode. More specifically, by changing different applied voltages to control “ON” and “OFF” states of the diodes, the desirable responses across the programmable metamaterial hologram can be achieved in an inexpensive and dynamic way. As such, a single information metamaterial hologram can accomplish various functions dynamically via FPGA.

The other key issue to realize the programmable holographic imaging is to calculate the 1-bit coding patterns corresponding to target images efficiently. Physically, the hologram has a linear relation to the current induced over the metamaterial illuminated by plane waves. In the conventional metamaterial hologram, the hologram and induced current are continuous functions and hence the Gerchberg-Saxton (GS) algorithm can be used to determine the holograms. In the digital coding metamaterial, however, the hologram must be a binary function. Therefore, the GS algorithm has to be modified for generating the binary phase profile of the reprogrammable hologram (Li et al., 2017). In fact, the design of the programmable hologram is mathematically casted into a combinatorial optimization problem, in which the back/forward propagation operations have to be considered carefully. As examples, Figures 10B and 10C report a set of experimental holographic images of a sentence of “LOVE PKU! SEU! NUS!”, in which the binary coding patterns of metamaterial are presented in Figure 10B. In experiments, we use FPGA to control the PIN diodes in the parallel way, in which the FPGA clock rate is 100 MHz, corresponding to the time of each operation cycle of 10 ns. To change the PIN diode status in parallel, three operation cycles are needed after compiling. Thus the total hologram reconfiguration time is around 33 ns. We remark that the programmable metamaterial hologram can be readily extended to exhibit multiple digital bits for both phase and amplitude modulations, which will lead to more versatile systems with adaptive and rewritable functionalities.

#### Direct Transmission System of Digital Message

Digital wireless communication system has experienced rapid development in the past 30 years and has become indispensable in daily life. In the currently used wireless communication systems, the information to be sent is firstly digitized, then converted to analog, modulated, and finally transmitted to terminals through a series of modules including the digital-analog (D/A) convertor, modulator, demodulator, mixer, digital up convertor and digital down convertor, and expensive radio-frequency (RF) components. One of the most important processes in wireless communication systems is the signal modulation, where the digital bit stream is conveyed on the EM waves and physically transmitted to space. Some widely used modulation techniques include amplitude-shift keying (ASK), frequency-shift keying (FSK), phase-shift keying (PSK), and quadrature amplitude modulation (QAM). They are designed to modulate the digital signal by changing the amplitude, frequency, and phase of the carrier wave. The modulated signal will then be up-shifted to the microwave frequency, amplified by a series of power amplifiers, and finally radiated to free space through antennas.

**Figure 11:**
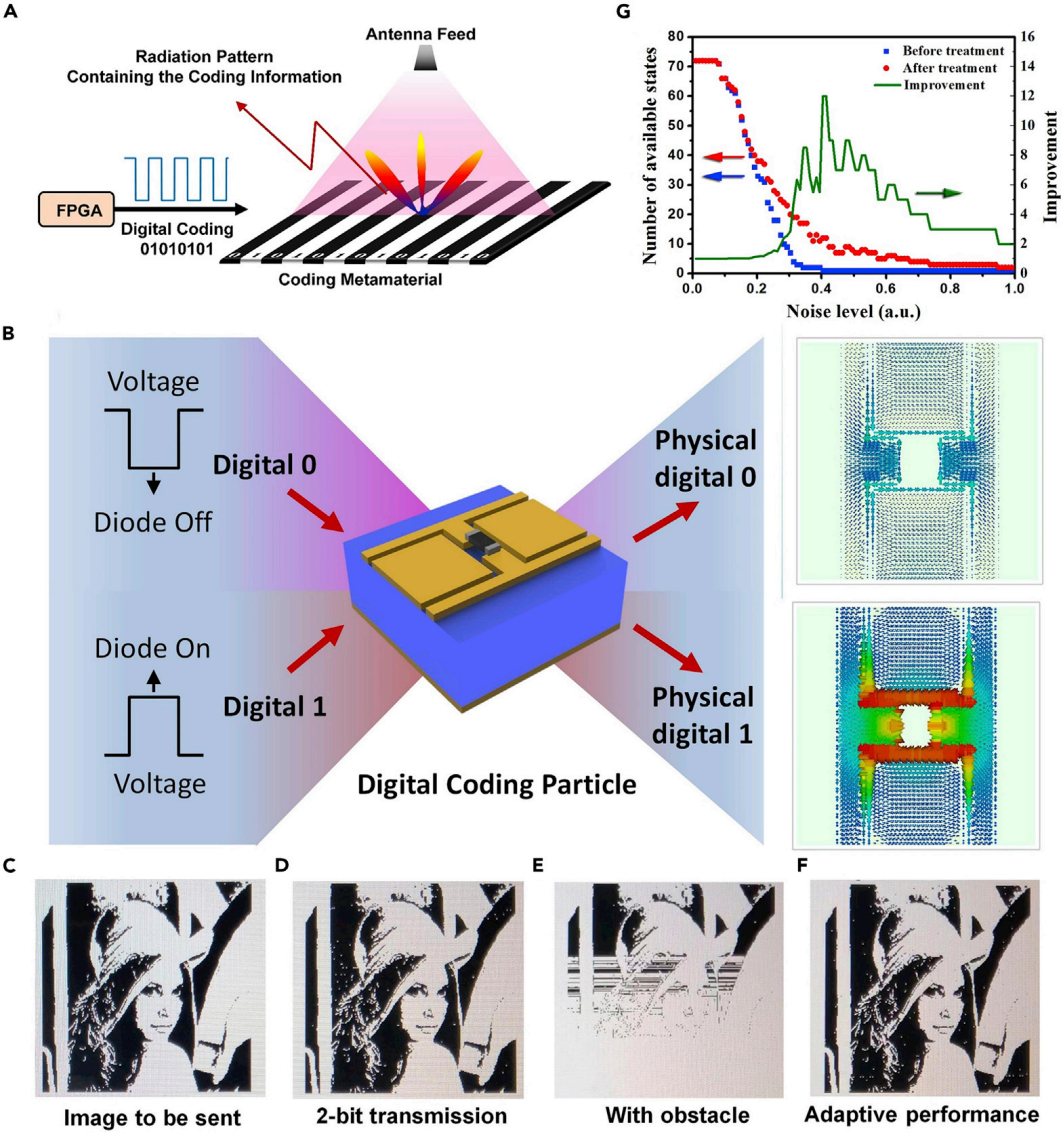
Directly Digital Modulation Based on the Programmable Metamaterial (A) Schematic illustration of the working mechanism of the proposed DDM system. (B) Meta-atom of the programmable metamaterials, which shows distinct current distributions and reflection phases under the 0 and 1 states. (C–F) Measurement results of the DDM wireless communication system. (G) The variation of numbers of the available digital states before and after the optimization treatment as the noise level increases from 0 to 1.

Because the programmable metamaterial provides dynamically changing radiation patterns corresponding to different digital information, the variation of far-field radiation patterns can be utilized in the modulation process for the wireless communication system (Cui et al., 2019). Based on this idea, we proposed a new digital wireless communication architecture based on the programmable metamaterial. As the signal is directly modulated by the dynamically changing radiation patterns and does not require the modules in the traditional wireless communication systems (e.g. mixers and D/A convertors), the system was also called as “directly digital modulation” (DDM) (Cui et al., 2019). Owing to the simplified system architecture of the DDM system in both hardware and software, the digitized signal can be directly loaded to the digital coding metamaterial and is sent to free space under the illumination of feeding antenna. As shown in Figure 11A, the digital information, being modulated in all possible radiation patterns of the metamaterial, can be correctly received by multiple receivers distributed in different locations in the far field region.

A prototype was fabricated to validate the new wireless communication system. Figure 11B demonstrates the meta-atom of the programmable metamaterial, where the digital bits “0” and “1” are converted to low and high voltages, respectively, to control the status of the PIN diode on each meta-atom of the metamaterial and change its reflection phases (Cui et al., 2019). The performance of the DDM system is evaluated by testing transmission of an image (see Figure 11C) under different conditions. The image was correctly received with two receivers in the 2-bit-symbol transmission mode, as shown in Figure 11D. To improve the transmission rate and system robustness against channel disturbance, a channel optimization algorithm is developed to allow the DDM system to function correctly in the presence of obstacles in the wireless channel. Figure 11E illustrates that severe transmission error occurs as soon as a metal plate is inserted between the transmitter and receivers. We could still achieve a correct image by using the channel optimization algorithm, which dynamically updates the best digital state for the optimum transmission (see Figure 11F). Figure 11G demonstrates the performance of the channel optimization algorithm in improving the number of available digital states for different noise levels. A four times improvement is achieved for most noise levels and could reach up to 12 at the noise level of 0.43. Most importantly, the new modulation technique can protect the information from being intercepted from either a single or multiple positions, making it promising for secrete communications.

### Intelligent Metamaterial Systems

Nowadays, various computational imagers have been proposed to fundamentally refresh the notation of EM sensing systems. However, these sensing devices are posing challenges including the low efficiency in data acquisition and the expensive cost in data processing. In these scenarios, there is a great urge to avoid massive data collection and acquire as few measurements as possible, and meanwhile the system is still able to recover the image and identify enough information of interests. To tackle the “data crisis” posed by the sheer size of raw data acquired and processed by the data sources, several contributions have emerged to overcome the formidable challenges by merging the programmable metamaterials and *artificial* intelligence together (Li et al., 2019a, 2019b).

#### Machine-Learning-Driven Real-Time Microwave Imager

Here we introduce a real-time microwave imager based on programmable metamaterial driven by machine learning algorithm (Li et al., 2019a, 2019b). Recent advances in machine learning state that high-quality imaging and high-accuracy object recognition can be realized by the machine-learning-guided imagers with the remarkably reduced measurements if they are properly designed. However, the link between the EM imagers and the machine learning techniques is still lacking. Specifically, almost all EM imagers cannot produce the radiation patterns required by the machine-learning-desired measurement modes in real time. As discussed earlier, the programmable metamaterial can be used to produce the desirable and complicated radiation patterns. Then, the programmable metamaterial can be well trained with the machine learning technique when the training samples are available. In this way, we can obtain the machine-learning-desired radiation patterns and realize multiple functions controlled by software without any hardware modifications, which is referred to as ***programmable imager***. We demonstrated that a 2-bit programmable metamaterial can produce the radiation patterns desired by the machine learning, like the principle component analysis (PCA) (Li et al., 2019a, 2019b). With such an imager, the number of measurements can be drastically reduced, leading to the real-time EM sensing for complicated scenes.

**Figure 12:**
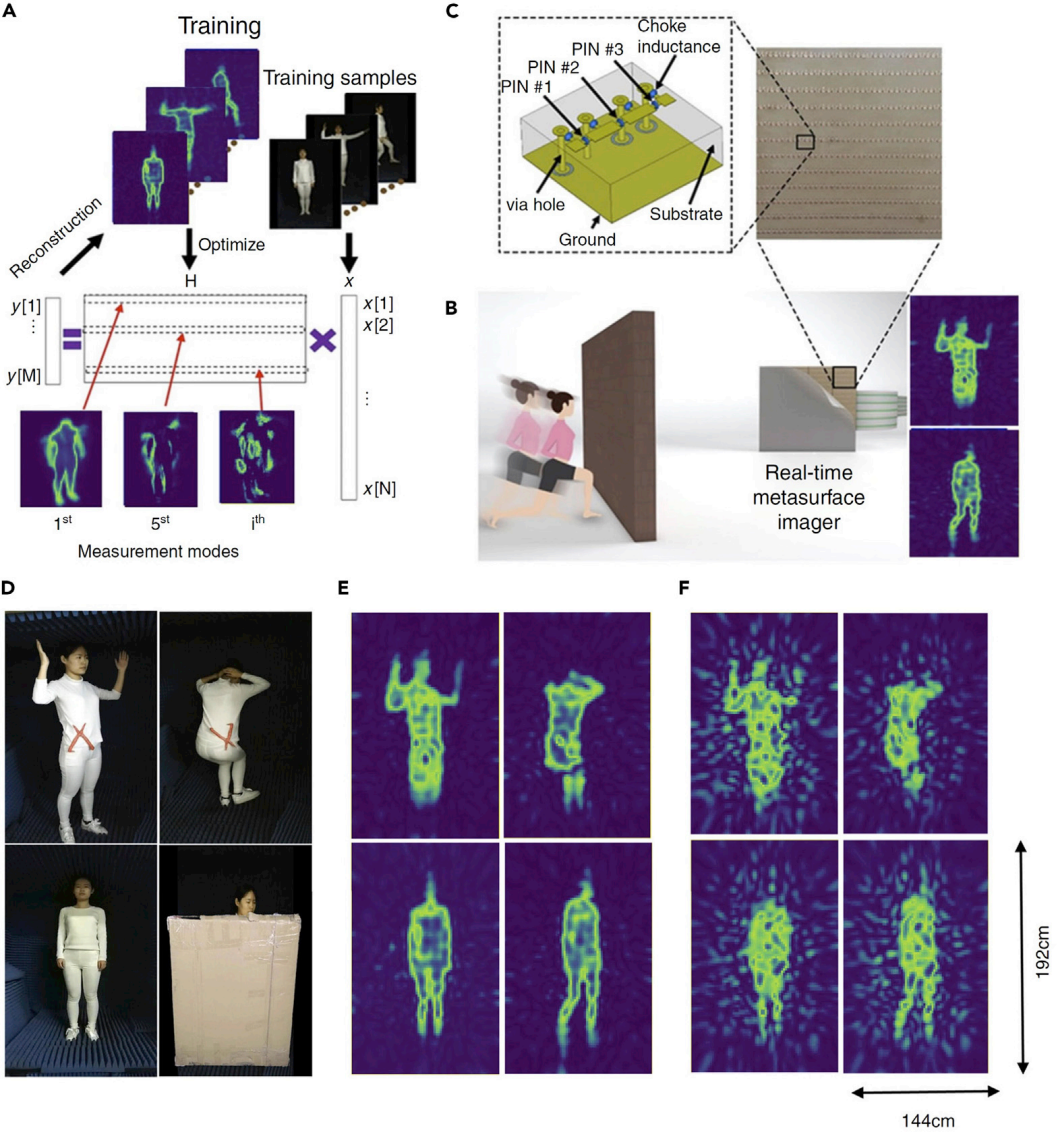
Principle of the Machine-Learning-Driven Microwave Imager and Experimental Results (A) The proposed machine-learning metamaterial imager is optimized by the training samples. The scene ***x*** is compressed through the matrix H: ***y*** = **H*x***. By minimizing the difference between the reconstructed scenes and the original scenes, the optimized matrix H is determined. (B) Photo of a 2-bit programmable metamaterial. (C) The sketch of a meta-atom. (D) Four cases of experimental tests. (E and F) The experimental results by using the developed imager trained with PCA and random projection, respectively. Reference: L. Li, NC (2019)

Figures 12A and 12B demonstrate the principle behind the programmable imager that can produce high-quality imaging and high-accuracy object recognition operating on the compressed measurements directly from the imager without making hardware alternation and computational-cost image reconstruction. The proposed microwave imager is based on a 2-bit programmable metamaterial (Li et al., 2019a, 2019b). Figure 12C shows the sketch of the designed 2-bit meta-atom, which consists of three PIN diodes and possesses one of the 2-bit phase responses (i.e., 0, π/2, π, and 3π/2) that satisfies the full 2π phase coverage by properly controlling the applied bias voltages of the PIN-diodes via FPGA. Based on the 2-bit coding metamaterial, the programmable imager enables not only real-time and high-quality machine-learning-guided imaging from significantly reduced measurements but also real-time object recognition directly in the compressed measurement domain without changing the hardware.

For illustration purpose, two popular linear embedding techniques of machine learning are considered, i.e., the random projection and the principal component analysis (PCA), to train our machine-learning imager (Li et al., 2019a, 2019b). Note that it is trivial to conceive a random projection matrix by independently and randomly setting the status of PIN diodes of the programmable metamaterial. However, for the PCA measurements, the status of PIN diodes in the programmable metamaterial need to be carefully manipulated to achieve the desirable measurement modes. Figure 12D gives four images of human bodies to be imaged, where the top two persons are armed with the toy of glass scissor, as marked in red. Figures 12E and 12F are the corresponding reconstructed images by using the machine-learning imagers trained with PCA and random projection, respectively, where 400 measurements are used. The measured results show that not only the gestures of the test person can be recovered by the machine-learning imager in real time but also the armed glass scissor can be clearly reconstructed, even when the people is behind an opaque wall. Moreover, we can see clearly from this set of figures that since a large amount of relevant training samples are incorporated in PCA, the reconstructions by PCA have overwhelming advantages over those by the random projection in cases of small amount of measurements. To summarize, our programmable imager could pave the avenue for future compressed imaging applications in microwave, millimeter wave, and terahertz frequencies, and beyond.

#### Deep-Learning-Driven Intelligent Microwave Camera

**Figure 13:**
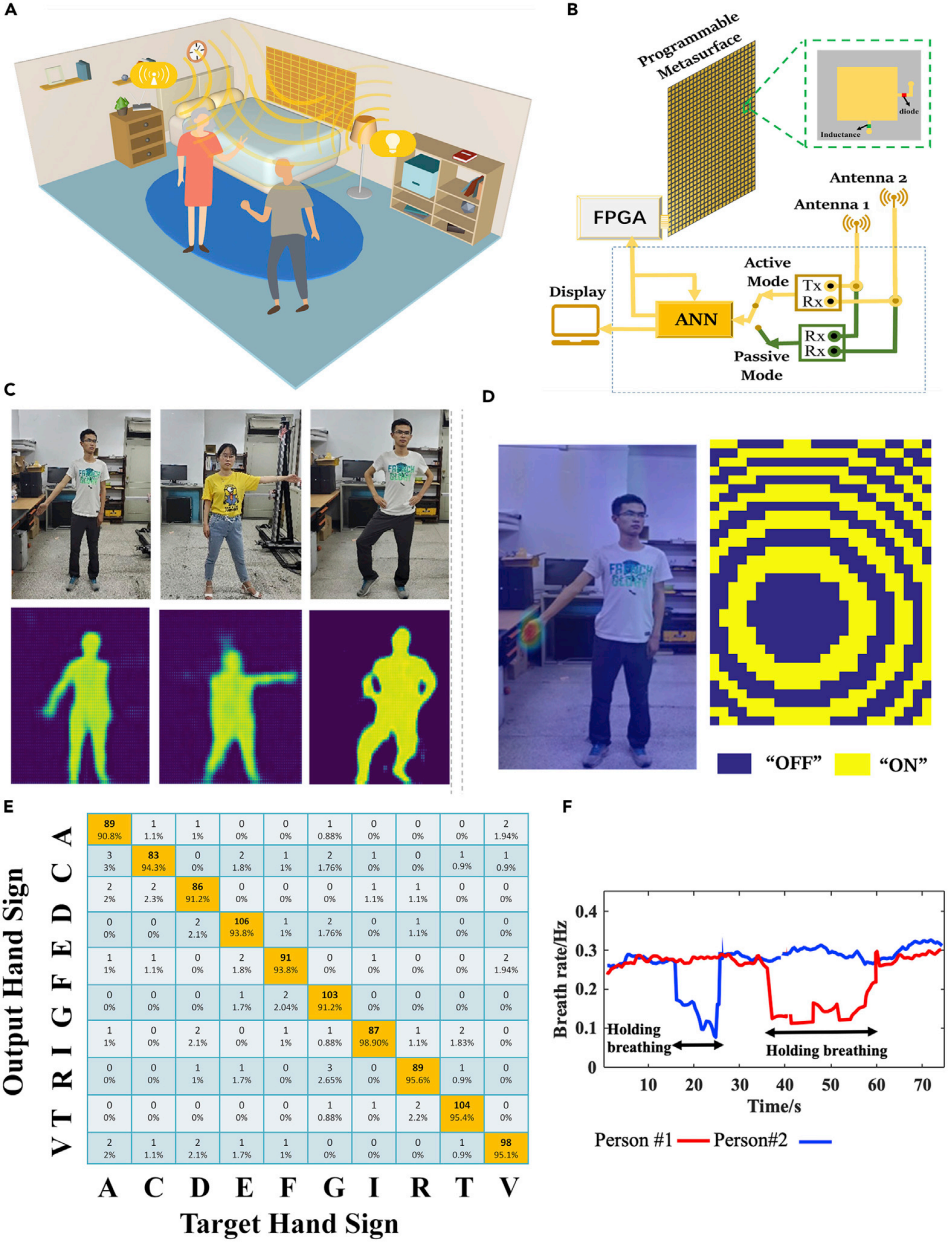
Principle of the Intelligent Microwave Camera (A and B) An illustrative scenario for monitoring people in a typical indoor environment in a smart, real-time and inexpensive way, where the intelligent metamaterial decorated as a part of wall is used to adaptively manipulate ambient Wi-Fi signals. (C) *In-situ* imaging results. (D) The left panel is the result of the Wi-Fi signals focused at the desirable spot of human hand, and the right panel is the corresponding coding pattern of the programmable metamaterial. (E) The classification matrix of 10 hand signs. (F) Results of human respiration of two non-cooperative persons behind a 5-cm-thickness wall. Reference: L. Li, Light (2019)

The Internet of Things (IoT) and Cyber Physical Systems (CPS) have opened up possibilities for smart cities and smart homes, which are changing the way people live. In this era, it is demanded to remotely probe where people are, what they are doing, what they want to express by their body language, and how their physiological states are, in a way not to infringe on visual privacy. By now, various radio-based contactless sensing devices have been developed; however, they are hardly deployed in real-world settings because they require the objects of interest to either deliberately cooperate or carry a wireless active device and/or identification tag. To address these limitations, we proposed the concept of intelligent microwave camera (Li, 2019) by integrating a series of convolutional neural networks (CNNs) for adaptively controlling data flow into the programmable metamaterial for adaptive data acquisition, as illustrated in Figure 13. Here, we would like to highlight three critical roles of the programmable metamaterial (see Figure 13B): (1) to fulfill *in-situ* high-resolution imaging of people in the full-viewing scene; (2) to rapidly focus the EM fields (including ambient Wi-Fi signals) to choose local spots and avoid undesired interferences from body trunk and ambient environment; and (3) to monitor the local body signs and vital signs of multiple non-cooperative people in real world by instantly scanning the local body parts of interest.

Three CNNs are developed in an integrated hierarchy, which are used to transform the measured microwave data into images of the whole human body, to classify the specifically designated spots (such as hand and chest) in the whole image, and to recognize the human hand signs in real time, respectively (Li, 2019). Here, the instantaneous *in-situ* imaging of the full scene and adaptive recognition of hand signs and vital signs of multiple non-cooperative people have been demonstrated (see Figure 13A). More interestingly, we show that the intelligent microwave camera works well even when it is passively excited by the stray 2.4GHz commodity Wi-Fi signals that ubiquitously exist in our daily lives.

Figures 13C–13F present a set of experimental results of *in-situ* imaging, hand-sign recognition, and respiration identification using the proposed intelligent microwave camera excited with the commodity stray Wi-Fi signals, where the subject person is behind a 5-cm-thickness wooden wall in indoor lab environment (Li, 2019). Based on the high-resolution images of the full human body, one can further realize a sequence of high-level recognition tasks, i.e., the recognition of hand signs and vital signs, which are accomplished by adaptively performing a three-step routine procedure in the active mode. Firstly, the Faster R-CNN is applied to process the full-scene image in order to instantly find the location of hand or body chest. Secondly, the digital coding patterns of the programmable metamaterial are optimized and configured through FPGA so that the stray Wi-Fi signals are spatially focused and enhanced on the desired spots. Finally, IM-CNN-2 or the time-frequency analysis algorithm is performed to realize the recognition of hand signs or vital signs. Figure 13D shows the results of the commodity Wi-Fi signals after being well focused at the desired location, e.g., the right hand of the subject person. Figures 13E and 13F illustrate the experimental results of the hand-sign and respiration recognitions of two people, which have better accuracies of 90% and 92%, respectively. By integrating the programmable metamaterial with artificial neural networks as a whole system, we believe that the concept of intelligent metamaterial can be extended over the entire EM spectra, which could open a promising avenue for future smart home, human-device interactive interface, health monitoring, safety screening, and intelligent sensing (Li et al., 2020).

### Space-Time-Coding Digital Metamaterial Systems

In the earlier discussions, the digital coding is defined in the spatial domain. We demonstrated that the space-coding sequences or patterns have been successfully used in controlling the spatial distributions of EM fields, such as the scattering beams or radiation beams. Here, we extend the digital coding from the spatial domain to time domain and show that time-domain digital coding metamaterials have powerful abilities in manipulating the spectral distributions of EM fields. Furthermore, the general space-time-coding digital metamaterial is presented to tailor the spatial distributions and spectral distributions simultaneously.

#### Time-Domain-Coding Digital Metamaterial

**Figure 14:**
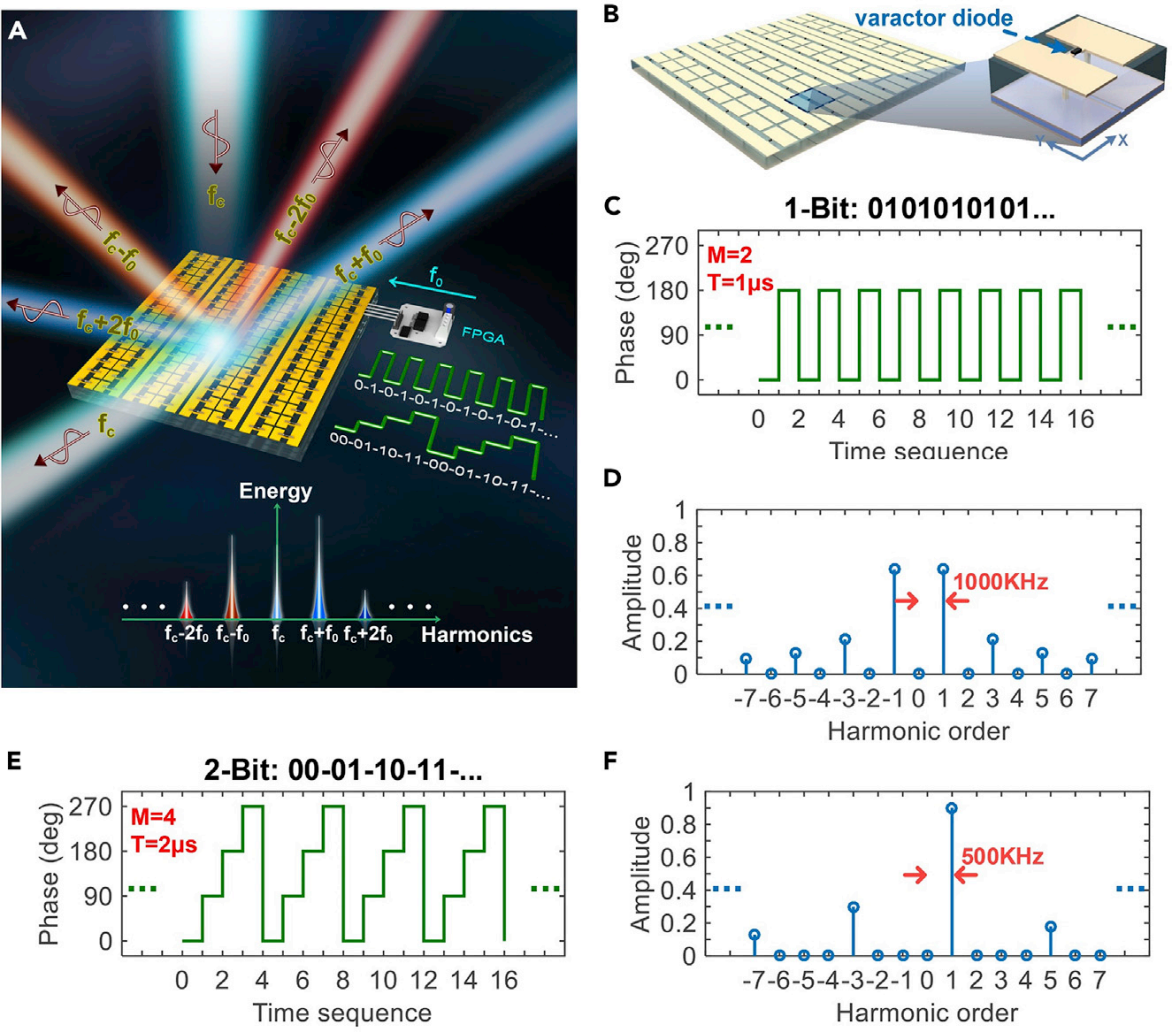
Independent Control of Harmonic Amplitudes and Phases via a Time-Domain Digital Coding Metasurface (A) Schematic of the time-domain-coding digital metamaterial and its control to EM spectra. (B) The meta-atom of metamaterial. (C) The 1-bit time coding sequence 01010101 … at 3.6GHz with the pulse duration τ = 1.6 μs. (D) The measured spectral intensities of the harmonics under the 1-bit coding sequence. (E) The 2-bit time coding sequence 00-01-10-11- … at 3.6GHz with the pulse duration τ = 1.6μs. (F) The measured spectral intensities of the harmonics under the 2-bit coding sequence.

We proposed a time-domain-coding digital metamaterial with the dynamic and programmable responses to modulate the local reflection features, which can be used to control the nonlinearity of EM waves with much stronger capability (Zhao et al., 2019), as shown in Figure 14A. In contrast to the space-domain coding metamaterials investigated previously, complex modulation strategies could be employed to tailor the wave-matter interactions of the fundamental and high-order harmonic waves simultaneously, where the discrete reflection phase states of meta-atoms are altered in time in the programmable way. The nonlinear process takes place by the temporal modulation of waves incident on the metamaterial. Under the monochromic incidence at frequency $f_{c}$, if the reflection coefficient of the metamaterial is driven as a periodic function Γ(t), then the reflected field in the frequency domain can be written as follows through the Fourier transform (Zhao et al., 2019)

(Equation 6) $$E_{r}\left(f\right)=E_{i}\left(f\right)\text{∗}\Gamma \left(f\right)=\delta \left(f-f_{c}\right)\text{∗}\sum_{k=-\infty }^{\infty }a_{k}\delta \left(f-kf_{0}\right)=\sum_{k=-\infty }^{\infty }a_{k}\delta \left(f-kf_{0}-f_{c}\right)$$

where δ(f) is the Dirac delta function, $f_{0}=1/\text{T}$ is the fundamental harmonic frequency, T is the period of Γ(t), and $a_{k}$ is the coefficient of the $k^{th}$-order harmonic. By elaborately designing the waveform of Γ(t), arbitrary harmonic distributions can be achieved theoretically. This time-varying feature allows accurate amplitude and phase controls of the harmonic waves, thus conveying the incident energy to higher-order harmonics.

Figure 14B shows a typical time-domain-coding digital meta-atom, in which the loaded varactor diode can be tuned under the biased voltage. Driven by external programmable devices such as microprogrammed control unit (MCU) and FPGA, the single metamaterial is able to operate with different functionalities by changing the coding states of all coding units. Figures 14C–14F present the measured spectral intensities under 1-bit and 2-bit time coding sequences. It is clear that the time-domain-coding digital metamaterial has powerful capabilities to manipulate the harmonic amplitude distributions by taking various coding strategy. In the meantime, it can also regulate the harmonic scattering patterns freely by introducing spatial phase discontinuities among the meta-atoms at the same time (Dai et al., 2018). The proposed theory and method will pave the way for designing simplified and compact communication and radar systems across the wavelength ranging from acoustic, microwave to optical regimes.

#### Time-Domain Convolution Theorem

**Figure 15:**
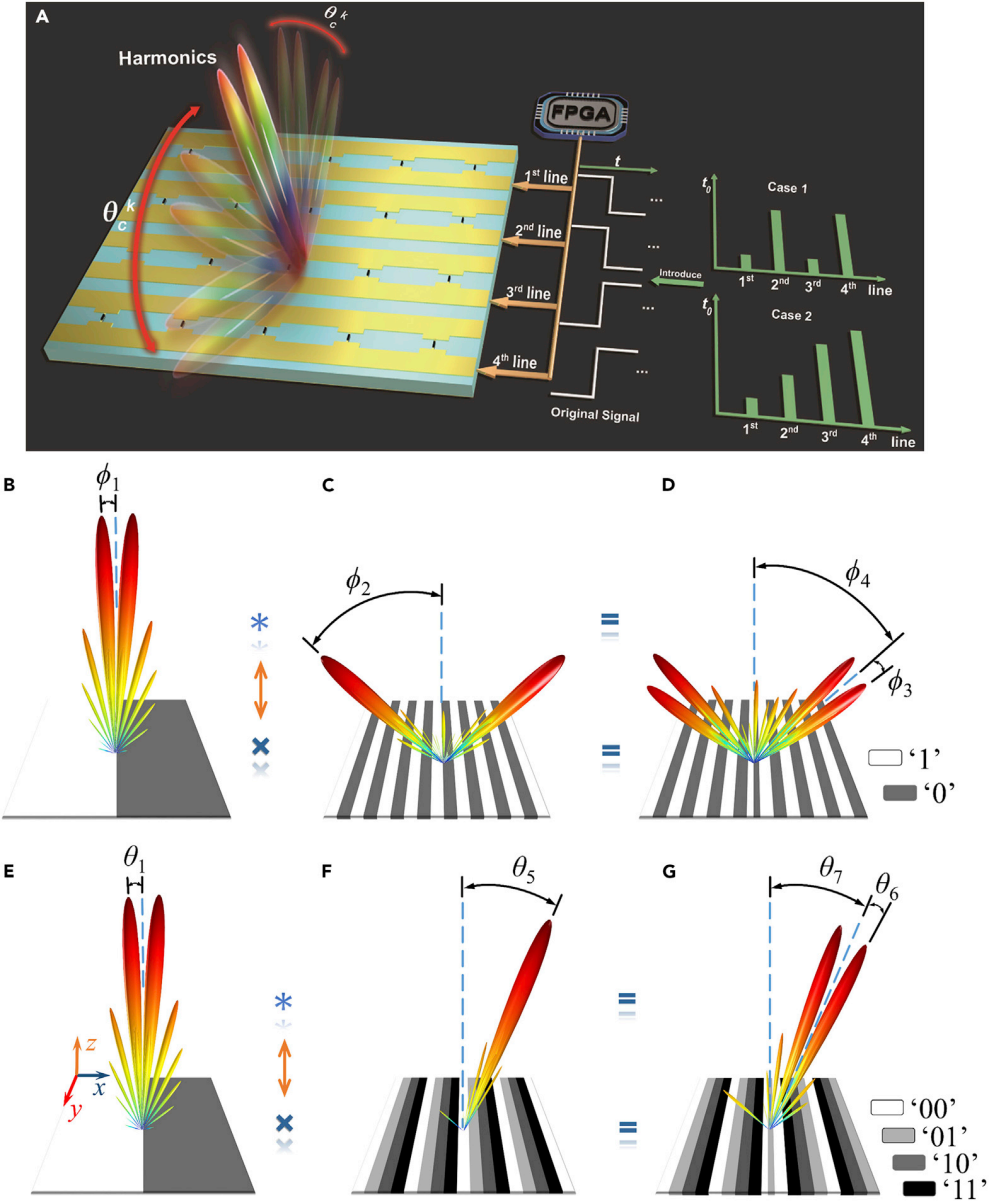
Convolution Operations on Time-Domain Digital Coding Metasurface for Beam Manipulations of Harmonics (A) Illustration of nonlinear convolution operations on harmonics to realize dual-beam scattering to arbitrary directions in the upper space. (B–D) 1-bit case for nonlinear convolution operation. (E–G) 2-bit case for nonlinear convolution operation.

With the aid of time-domain-coding digital metamaterial, some prominent nonlinear effects have been achieved, including frequency shifting, harmonic generation, and optical isolation. However, up to now, flexible and accurate manipulations of the harmonic waves still lack a systematic research. Therefore, we propose a nonlinear time-domain convolution theorem to address this problem based on the Fourier transform pair between the coding pattern and far-field pattern (Zhang et al., 2020c), as illustrated in Figure 15A. This is a counterpart of the convolution theorem for the space-domain-coding metamaterial discussed in the earlier section.

It is known that when the reflection coefficient is delayed by $t_{0}$ in time, the corresponding reflection spectrum can be written as

(Equation 7) $$\text{Γ}\left(t-t_{0}\right)↔e^{-j\omega t_{0}}\text{Γ}\left(\text{jω}\right)$$

When the metamaterial works at the frequency of $\omega_{0}$, the *k*^*th*^-order harmonic at $k\omega_{0}$ will be attached by an additional phase $e^{-jk\omega_{0}t_{0}}$ without changing the reflection amplitude. Therefore, it is possible to efficiently adjust the harmonic phase of each meta-atom by independently altering the time shift $t_{0}\left(x\right)$ of the controlling circuit, where *x* is the element position. By introducing abrupt phase changes among the meta-atoms, anomalous reflections for the *k*^*th*^-order harmonic can be realized, in which the reflection angle $\theta_{c}^{k}$ is determined by (Zhang et al., 2020c)

(Equation 8) $$\theta_{c}^{k}=\arcsin\left(\frac{\lambda^{k}}{2\pi }\frac{d\left(-k\omega_{0}t_{0}\left(x\right)\right)}{dx}\right).$$

where $\lambda^{k}$ is the corresponding operation wavelength. It is clear that $d\left(-k\omega_{0}t_{0}\left(x\right)\right)/dx$ plays the same role as dΦ/dx in the generalized Snell's law (Yu et al., 2011) to provide the necessary phase gradient for bending harmonic waves into arbitrary directions. Under pre-set time shifts on the meta-atom, arbitrary wave front can be generated in continuous according to Equation (8). For the convenience of design and implementation, we use discrete phase states instead of continuous phase distribution to construct the n-bit metamaterial. From the convolution theorem (Liu et al., 2016a, Liu et al., 2016, 2016c, 2016d, 2016e), we can add the primary and secondary digital coding patterns to bend the *k*^th^-order beam at the desired direction. This is the time-domain convolution theorem.

To verify this theory, two different examples are provided. In the first example (see Figures 15B–15D), we choose the delay time $t_{0}$= 0 or *T*/2 to construct the 1-bit time-domain digital state “0” (white color) or “1” (dark gray color) for odd-order harmonics. When the primary coding sequence S1 “0 0 … 1 1 … ” and the secondary coding sequence S2 “0 0 1 1 0 0 1 1 … ” are added together, the mixed time-domain coding sequence is obtained in Figure 15D. From the scattering patterns, it is clear that the original two beams of the first harmonic are deviated from the normal axis by $\theta_{2}$ without amplitude distortion, which is consistent with the convolution theorem. To generate asymmetric harmonic scattering patterns, Figures 15E–15G show results of the second example with 2-bit time-domain-coding digital metamaterial.

#### New-Architecture Wireless Communication Systems

**Figure 16:**
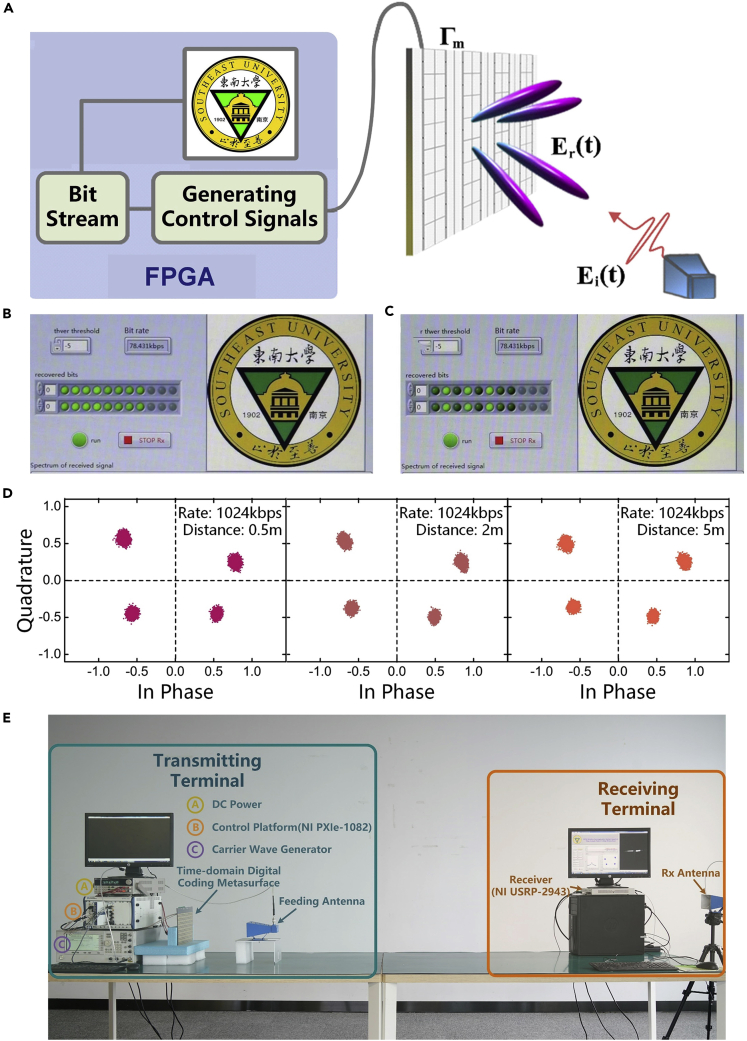
New-Architecture Wireless Communication Systems Based on Time-Domain-Coding Digital Metamaterial (A) Schematic of the BFSK wireless communication system based on the time-domain-coding digital metamaterial. (B and C) The received messages (the image with Southeast University Logo) by the BFSK wireless communication system for different receiving angles α=0° and 30°, respectively. (D) The dependence of the measured constellation diagrams on the message transmission rate and communication distance at the receiving terminal. (E) The measured movie transmission.

The above analyses show that the time-domain-coding digital metamaterial has powerful ability in controlling the amplitude and phase spectra of the reflected signals simply and accurately (Zhao et al., 2019; Dai et al., 2018; Zhang et al., 2020c), which allows the information modulation process to be performed at the metamaterial interface. Such properties are especially promising for realizing directional signal modulations, because the baseband signals can be directly modulated on the carrier waves without the D/A conversion and mixing process. According to the mapping relationship between the baseband information and control signals of metamaterial, the new wireless communication system support various modulation schemes including ASK, FSK (Zhao et al., 2019), PSK (Dai et al., 2019; Tang et al., 2019a; 2019b), and even high-order modulation schemes such as QAM (Dai et al., 2020). For example, Figure 16A demonstrates the schematic of a binary FSK (BFSK) wireless communication system based on the time-domain-coding digital metamaterial (Zhao et al., 2019). The corresponding experimental scenario and measurement results are illustrated in Figures 16B and 16C, respectively, in which an image is sent successfully to the receiver with the data transmission speed of 78.125 kbps at carrier frequency 3.6 GHz. To improve the data transmission speed, a new quadrature PSK (QPSK) wireless communication system was further presented (Dai et al., 2019). In this case, the data transmission speed reaches 1 Mbps at the carrier frequency of 4 GHz, guaranteeing smooth transmission of a movie without any interuption (Dai et al., 2019). The measured constellation diagrams at the receiving terminal with different message transmission rates and communication distances are plotted in Figure 16D, and the measured movie transmission is illustrated in Figure 16E. Both prototypes are able to realize real-time data transmissions, and worked well in indoor scenarios, proving the accuracy and reliability of developed systems. This novel architecture of wireless communication system may play important role in the future wireless technologies.

#### Space-Time-Coding Digital Metamaterial Systems

As we have discussed previously, the space-domain digital metamaterials can control the spatial distributions of EM fields, whereas the time-domain digital metamaterials control the spectral distribution of EM fields. Here, we further introduce the concept of space-time-coding digital metamaterials (Zhang et al., 2018a, 2018b, 2019a, 2019b), which are digitally encoded in both the space and time domains. Each digital meta-atom is temporally modulated with a periodic time-coding sequence. Together with spatial modulations, the space-time-coding digital metamaterial can be used to control both spatial and spectral characteristics of EM fields. By suitably designing the space-time-coding matrix, the digital information can be encoded and processed not only in the space domain but also in the time domain. The space-time-coding strategy significantly extends the application scope of the programmable metamaterials, leading to important applications in the wireless communications and radar systems. Since the space-time-coding digital metamaterial was initially proposed in 2018 (Zhang et al., 2018a, 2018b), it has been successfully applied in many applications including harmonic beam steering, beam shaping, scattering-signature control, wave-based frequency conversion, nonreciprocal effect (Zhang et al., 2019a, 2019b), and multi-bit programmable phase generations (Zhang et al., 2019a, 2019b). Compared with the traditional space-time metamaterials based on analog modulations (Hadad et al., 2015, 2016; Shaltout et al., 2015; Correas-Serrano et al., 2016), the space-time-coding digital metamaterial provides an easier fabrication and more powerful capabilities.

**Figure 17:**
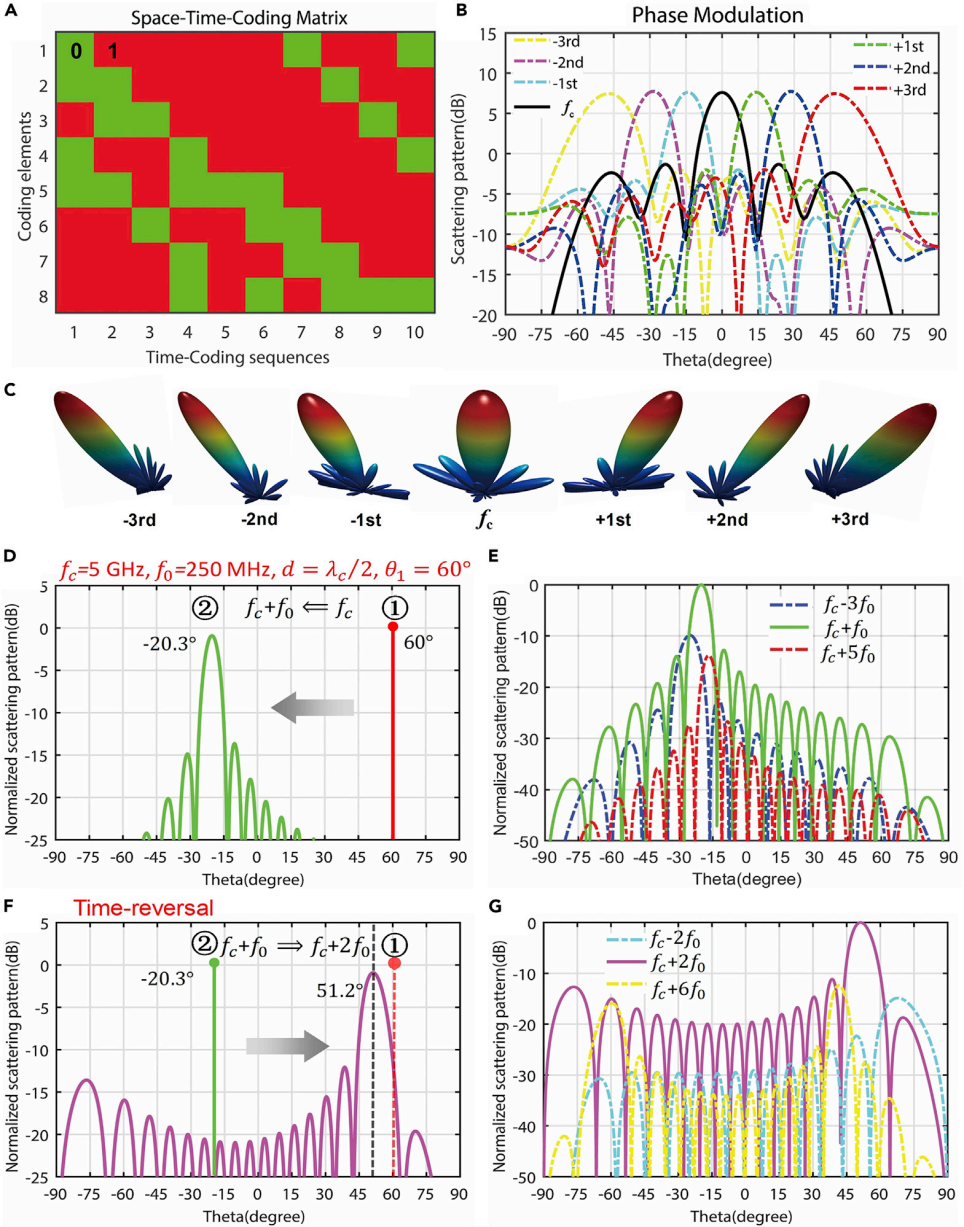
Realizing Harmonic Beam Steering Using a Binary Particle Swarm Optimization (BPSO) Algorithm (A–C) Harmonic beam steering based on the space-time-coding digital metamaterial. (A) The BPSO-optimized space-time-coding matrix. (B and C) The corresponding scattering-pattern cuts at ϕ = 90°, and 3D scattering patterns at different harmonic frequencies. (D–G) Nonreciprocal reflection effects enabled by the space-time-coding digital metamaterial. (D and E) Scattering patterns in the forward scenario (excitation from port 1 at $f_{c}$ and $\theta_{i}=60{}^{\circ}$). (F and G) Scattering patterns in the time-reversal scenario (excitation from port 2 at $f_{c}+f_{0}$ and $\theta_{i}=20.3{}^{\circ}$).

We discuss two representative examples to illustrate the working principles and advantages of the space-time-coding digital metamaterial. The first example aim at realizing harmonic beam steering using a binary particle swarm optimization (BPSO) algorithm. According to the general theory of space-time-coding metamaterial (Zhang et al., 2018a, 2018b), the scattering patterns can be precisely controlled at any harmonic frequencies. To achieve the beam steering at different harmonics, we use the BPSO algorithm and define a specific fitness function to generate 1-bit space-time-coding matrix, as shown in Figure 17A. The corresponding harmonic scattering patterns are presented in Figure 17B, from which we can see that the main beams at different harmonic frequencies point to different spatial directions with nearly the same intensity, thereby realizing the desired harmonic beam steering. Figure 17C displays the corresponding 3D scattering patterns. All results show that the space-time-coding digital metamaterial with suitable coding matrix can successfully attain the harmonic beam steering, which has the feature of controlling the spatial distributions of EM powers at different harmonic frequencies.

The second example is to realize nonreciprocal effect based on a 2-bit space-time-coding digital metamaterial. As we know, space-gradient phase metamaterials are inherently constrained by Lorentz reciprocity, implying that a reflected wave in the time-reversed case will propagate along the same direction as the original incidence with the same frequency. But the space-time-coding digital metamaterial can break the reciprocity (Zhang et al., 2019a, 2019b). By inducing suitable space-time phase gradients in a programmable way, the nonreciprocal effects are controlled dynamically. Here we illustrate the nonreciprocal reflection effect based on 2-bit space-time-coding metamaterial, in which the forward and time-reversal reflection scenarios are taken into consideration. The programmable metamaterial is modulated by a specially designed space-time-gradient coding matrix. For the forward scenario, a plane wave with frequency $f_{1}$ at the incident angle $\theta_{1}$ is anomalously reflected at frequency $f_{2}$ with angle $\theta_{2}$. But for the time-reversal scenario, the incident plane wave with frequency $f_{2}$ at angle $\theta_{2}$ is reflected at the frequency $f_{3}$ with angle $\theta_{3}$. Due to the space-time-coding modulation, the frequency $f_{3}$ and angle $\theta_{3}$ differ from the original frequency $f_{1}$ and angle $\theta_{1}$, i.e. $f_{3}\neq f_{1}$ and $\theta_{3}\neq \theta_{1}$, which obviously breaks the time-reversal symmetry and Lorentz reciprocity. Specifically, Figures 17D and 17E present the scattering patterns at various harmonic frequencies for the forward scenario. The plane wave with frequency $f_{c}$ from port 1 incident on the space-time-coding metamaterial at angle $\theta_{1}=60^{\circ }$ produces a dominant beam at port 2 at angle −20.3° with frequency $f_{c}+f_{0}$. When considering the time-reversal case, the plane wave with frequency $f_{c}+f_{0}$ from port 2 incident at angle $\theta_{2}=20.3{}^{\circ}$ produces a reflection beam at angle $\theta_{3}=51.2{}^{\circ}$ with frequency $f_{c}+2f_{0}$, as shown in Figures 17F and 17G. We note that the angle $\theta_{3}$ and frequency $f_{c}+2f_{0}$ of the time-reversal reflected wave are different from the incident angle $\theta_{1}$and frequency $f_{c}$. There exists spatial separation and frequency isolation between the forward channel and time-reversal channel, demonstrating that the space-time-coding metamaterial can break the time-reversal symmetry and the Lorentz reciprocity in both space and frequency domains. It should be mentioned that the space-time-coding digital metamaterial can realize the nonreciprocal effect in programmable fashions, which not only produces some novel physical phenomena but may also find many promising applications in communications, heat management, energy harvesting, frequency conversion, Doppler frequency illusion, and optical isolation.

## Self-Adaptively Smart Metamaterials

In the earlier section, we intensively introduced various kinds information metamaterial systems. However, no matter in the traditional tunable and reconfigurable metamaterials or in the newly developed reprogrammable and even software/intelligent metamaterials, human beings must participate in the control actions to tune and switch the active devices or to send the instructions to FPGA. The reason is that all abovementioned metamaterials are open-loop systems and do not contain sensing and feedback components to establish a closed-loop system for automatic decision-making. To solve this problem, a sensor must be integrated into the software/intelligent metamaterial to create a self-adaptively smart metamaterial (Ma et al., 2019), as illustrated in Figure 1.

The smart metamaterial is based on the programmable metamaterial but consists of sensing and feedback components, which help constitute a smart closed-loop system for the metamaterial without the need of manual instructions. The sensor can detect specific features of metamaterial and its environment (e.g. spatial attitude and movement status) and send the feedback to FPGA in real time. Then the metamaterial will realize the self-adaptively reprogrammable functions automatically based on the on-site calculations using FPGA (Ma et al., 2019). The automatic and self-adaptive behavior is the fourth stage of the metamaterial developments.

**Figure 18:**
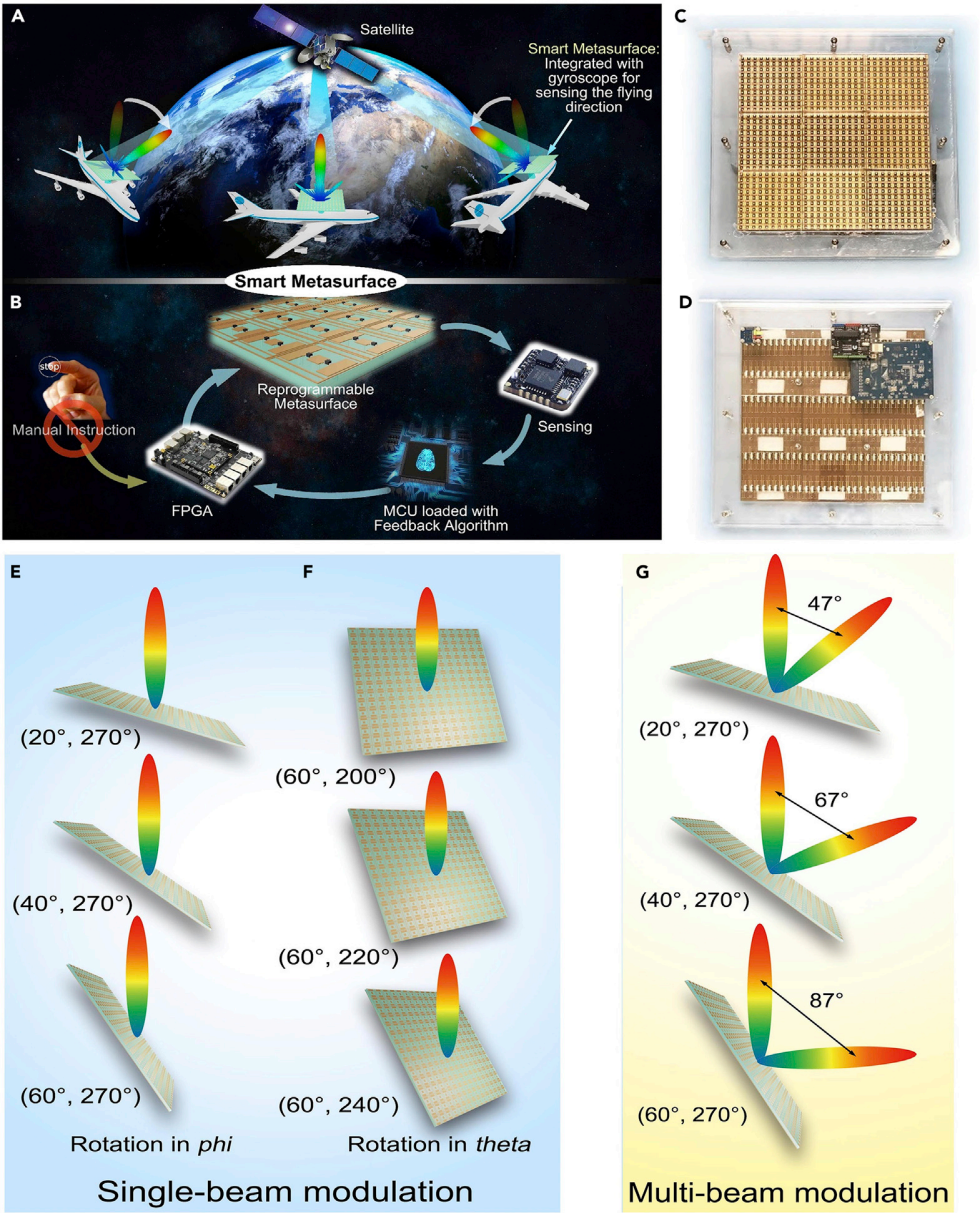
The Smart Metamaterial Mechanism and Its Typical Application (A) The application scenario in satellite communication. The smart metamaterial on the air-plane will automatically adjust its beam direction when the attitude changes. (B) The components of a smart metamaterial system. (C and D) The top and bottom views of the metamaterial sample. (E and F) The single-beam staring when the metamaterial rotates along the radial and normal directions. (G) The dual-beam modulations when the metamaterial rotates.

To specifically illustrate the application of smart metamaterial, we assume a typical scenario of satellite communication, as shown in Figure 18A. When an airplane flies around the earth, the radiation beam generated by the metamaterial needs to focus on the satellite all the time for steady communications (Ma et al., 2019). The smart metamaterial can sense the attitude variation as the airplane swerves and self-adaptively change the beam radiation direction. To achieve this mechanism, a sensing module and feedback algorithms are acquired on the programmable metamaterial, to construct a closed-loop system, as depicted in Figure 18B. FPGA, acting as a central brain, deals with the sensing data and executes the specific coding patterns according to the pre-arranged functions, which are usually carried out manually in the conventional metamaterials. Therefore, the smart metamaterial no longer needs external human instructions and can determine its reactions independently.

We designed and fabricated a smart metamaterial integrated with a gyroscope sensor and FPGA on the back of the programmable metamaterial. The top and bottom views of the fabricated sample are illustrated in Figures 18C and 18D, where all controlling lines for bias voltage are placed on the backside and are connected with FPGA. The gyroscope sensor and a microcontroller unit (MCU) are also linked to FPGA, to provide feedback for the rotating angle data in 3-axes. According to the rotating posture of metamaterial and on-site calculation algorithm, FPGA deploys the predesigned radiation patterns, which are stored in MCU (Ma et al., 2019).

To clearly illustrate beam manipulations when the metamaterial rotates, we provide single- and dual-beam modulation schemes. The main rotation directions can be classified as along the elevation and azimuth angles (phi and theta). For the beam-staring application, to keep the reflected beam aiming at the fixed target, the beam-scattering direction with respect to the metamaterial needs to change when the metamaterial rotates. Hence, the phase patterns on the metamaterial are equivalent to the beam deflection situations, assuming that the feed source rotates together. We provide two rotation schemes in Figures 18E and 18F, in which the scattering beam always stares at the north pole when the metamaterial rotates in phi and theta angles, respectively. In Figure 18E, as the theta angle is fixed at 270°, the phi angle of the metamaterial varies from 20° to 60°. Similarly, in Figure 18F, the theta angle sweeps from 200° to 240° when the phi angle is fixed to 60°. Besides, not limited to the single-beam modulation, we also achieved dual-beam modulations, in which each reflected beam is independently controlled. We consider the rotation in the phi direction, as shown in Figure 18G, in which one beam is programmed for staring, whereas the other executes beam scanning. When the metamaterial rotates, the scanning beam turns from 27° to 87°. Moreover, we further propose a fast algorithm to design the coding patterns (Ma et al., 2019), which can be easily carried out by MCU and FPGA. For single- or multi-beam modulations with desired scattering directions, the design algorithm can calculate the pattern configuration immediately. In realistic applications, such fast coding and programmable method can improve the response speed and remarkably reduce the cost and system complexity.

## Conclusion

In summary, during the past 24 years, the metamaterials have been well developed from theory to experiments, and further to applications, in controlling the EM waves, finding new physics, and developing useful devices. There have been many approaches to classify the metamaterials, such as in different space forms (3D and 2D) and in different frequency bands (microwave, terahertz, infrared, and optical). From the types of achievable functionalities, as we summarized in this article, the development of metamaterial are classified into four stages. The first stage is the structure-only passive metamaterial. The passive metamaterial has been shown very powerful in manipulating the EM waves. However, the function of passive metamaterial is fixed function at the time it is designed and fabricated. To realize more functions with a single metamaterial, the passive metamaterial must be integrated with an active component to reach the second stage of metamaterial: the active metamaterial, as shown in Figure 1. Usually there are two types of active metamaterials: tunable metamaterial and reconfigurable metamaterial. Tunable metamaterial can realize a single function with varied performance by tuning the active device, whereas the reconfigurable metamaterial can reach limited but significantly different functions by switching the active device at different states.

In order to achieve many significantly different functions with a single metamaterial, the digital coding characterization was proposed to introduce the digital states of metamaterial and control the EM waves by using digital coding patterns. When the digital metamaterial is equipped with FPGA, the metamaterial becomes field programmable, as illustrated in Figure 1, which can realize many significantly different functions in real time. This is the third stage of the development of metamaterials. We have mainly focused on the digital coding and programmable metamaterials in this review article. The digital coding representation of the metamaterial sets up a bridge between the physical world and digital world, and hence we can use not only the physical principles but also digital information methods to control both EM waves and digital information, leading to the appearance of information metamaterial. With this concept, the metamaterial is not limited to an effective material or a device but can be an information system. We have introduced the information metamaterial systems in detail, including the programmable metamaterial systems, the software metamaterial systems, the intelligent metamaterial systems, and the space-time-coding digital metamaterial systems.

However, in all abovementioned tunable, reconfigurable, and programmable metamaterials, human beings are required to the control actions to tune and switch the active devices or send the instruction to FPGA. The reason is that all of these metamaterials are open-loop systems and do not contain the sensing and feedback components that are required to establish a closed-loop system for automatic decision-making. Then we presented the self-adaptively smart metamaterial, which is based on the programmable metamaterial but consists of sensing and feedback components to constitute a smart closed-loop system without the need of manual instructions, as illustrated in Figure 1. This is the fourth stage of metamaterial developments. The smart metamaterial can achieve self-adaptively reprogrammable functions automatically using the sensing-feedback system and calculation software.

The self-adaptive metamaterial gets rid of human operations and can perform some actions automatically. However, the actions are still pre-designed and the on-site calculation algorithm is pre-written. That is to say, the self-adaptive metamaterial is still not equipped with the self-learning ability to define new functions. In the future, the self-adaptive metamaterials should be combined with a database that has a large number of environment images, functions, and the machine-learning algorithms to generate cognitive metamaterials (Cui et al., 2017; Cui, 2017, 2018; Liu and Cui, 2017; Ma and Cui, 2020) (see Figure 1), which could self-learn some new functions and operate in fully intelligent ways. This should be the fifth stage of metamaterial and many new technologies are expected to develop to reach the new stage.

Besides developing the cognitive metamaterial, there are many other directions that are worth to investigate on the information metamaterial. In the future, the suggested research topics include but are not limited to

• New approaches to control the digital states of meta-atoms, such as using MEMS, microfluid, and amplifier (Chen et al., 2019; Ma et al., 2019), besides the mainly used PIN diodes in the current stage.
• New degree of freedom to define the digital information, such as anisotropic coding (Liu et al., 2016a, Liu et al., 2016, 2018), polarization coding (Ma et al., 2017, 2020), frequency coding ([Bibr bib108][Bibr bib107]), and amplitude-phase coding ([Bibr bib109][Bibr bib110]), besides the mainly used space-domain phase coding and space-time-domain phase coding in the current stage.
• New information theory and signal processing methods on the information metamaterials, besides the information entropy and convolution theorem.
• New information systems based on the intelligent metamaterials for different application scenarios.

There are many possibilities and imaginations on the future of information metamaterials and their system applications.

We remark that all concepts introduced in this review are not limited to microwave but can be extended to high frequencies such as terahertz, infrared, and even visible light. Due to the smaller size of the digital units, new techniques should be employed to control their digital states. Two major ways can be employed to control these tiny units: electrical and optical approaches. The electrical way serves as the first choice due to the fast control on the digital state of the digital unit, which can be as small as a few nanosecond and is limited by the switching time of the diode. It also features compact size and light weight because all control devices can be fabricated on the same board with the programmable metamaterial. However, the electrical control poses great challenges on fabrication as the size of digital units decreases to a few tens of micrometers, for example, the complicated feeding lines and their bonding-wire connection with the external control unit, making it difficult to be extended to the infrared and visible lights. The optical control can be potentially employed when the frequency reaches this regime, for example, one can use spatial light modulator (SLM) to independently manipulate the conducting properties of photosensitive semiconductor materials. This approach simplifies the fabrication difficulties on the programmable metamaterials, allowing the size of the digital unit to be further shrinked to several micrometers or even down to nanoscales by immersing both SLM and metamaterials into high-permittivity liquid. However, the optical approach will gain larger size and weight of the entire system, due to the bulky size of the lens module.
